# Strain-dependent effects of clinical echovirus 30 outbreak isolates at the blood-CSF barrier

**DOI:** 10.1186/s12974-018-1061-4

**Published:** 2018-02-20

**Authors:** Tobias Dahm, Ortwin Adams, Sindy Boettcher, Sabine Diedrich, Vasily Morozov, Grant Hansman, Petra Fallier-Becker, Sebastian Schädler, Claus J. Burkhardt, Christel Weiss, Carolin Stump-Guthier, Hiroshi Ishikawa, Horst Schroten, Christian Schwerk, Tobias Tenenbaum, Henriette Rudolph

**Affiliations:** 1 Pediatric Infectious Diseases, University Children’s Hospital Mannheim, Medical Faculty Mannheim, Heidelberg University, Mannheim, Germany; 2 Institute of Virology, University Hospital, Heinrich-Heine-University, Düsseldorf, Germany; 3 National Reference Centre for Poliomyelitis and Enteroviruses, Robert Koch-Institute, Berlin, Germany; 4 Schaller Research Group, University of Heidelberg and the DKFZ, Heidelberg, Germany; 5 Department of Infectious Diseases, Virology, University of Heidelberg, Heidelberg, Germany; 6 Institute of Pathology and Neuropathology, University Hospital of Tübingen, Tübingen, Germany; 7 Carl Zeiss Microscopy GmbH, ZEISS Group, Oberkochen, Germany; 8 NMI Natural and Medical Sciences Institute, University of Tübingen, Reutlingen, Germany; 9 Institute of Medical Statistics and Biomathematics, Medical Faculty Mannheim, Heidelberg University, Mannheim, Germany; 10 Department of NDU Life Sciences, School of life Dentistry, The Nippon Dental University, Tokyo, Japan

**Keywords:** Enterovirus, Echovirus (E-30), Blood-cerebrospinal fluid barrier, Outbreak strains, Tight junction, Genomic sequencing, CNS, Meningitis, Transcellular transmigration, Paracellular transmigration

## Abstract

**Background:**

Echovirus (E) 30 (E-30) meningitis is characterized by neuroinflammation involving immune cell pleocytosis at the protective barriers of the central nervous system (CNS). In this context, infection of the blood-cerebrospinal fluid barrier (BCSFB), which has been demonstrated to be involved in enteroviral CNS pathogenesis, may affect the tight junction (TJ) and adherens junction (AJ) function and morphology.

**Methods:**

We used an in vitro human choroid plexus epithelial (HIBCPP) cell model to investigate the effect of three clinical outbreak strains (13-311, 13-759, and 14-397) isolated in Germany in 2013, and compared them to E-30 Bastianni. Conducting transepithelial electrical resistance (TEER), paracellular dextran flux measurement, quantitative real-time polymerase chain reaction (qPCR), western blot, and immunofluorescence analysis, we investigated TJ and AJ function and morphology as well as strain-specific E-30 infection patterns. Additionally, transmission electron and focused ion beam microscopy electron microscopy (FIB-SEM) was used to evaluate the mode of leukocyte transmigration. Genome sequencing and phylogenetic analyses were performed to discriminate potential genetic differences among the outbreak strains.

**Results:**

We observed a significant strain-dependent decrease in TEER with strains E-30 Bastianni and 13-311, whereas paracellular dextran flux was only affected by E-30 Bastianni. Despite strong similarities among the outbreak strains in replication characteristics and particle distribution, strain 13-311 was the only outbreak isolate revealing comparable disruptive effects on TJ (Zonula Occludens (ZO) 1 and occludin) and AJ (E-cadherin) morphology to E-30 Bastianni. Notwithstanding significant junctional alterations upon E-30 infection, we observed both para- and transcellular leukocyte migration across HIBCPP cells. Complete genome sequencing revealed differences between the strains analyzed, but no explicit correlation with the observed strain-dependent effects on HIBCPP cells was possible.

**Conclusion:**

The findings revealed distinct E-30 strain-specific effects on barrier integrity and junctional morphology. Despite E-30-induced barrier alterations leukocyte trafficking did not exclusively occur via the paracellular route.

**Electronic supplementary material:**

The online version of this article (10.1186/s12974-018-1061-4) contains supplementary material, which is available to authorized users.

## Background

Non-polio enteroviruses (NPEV) are the most common cause of viral meningitis in the developed world especially in children [1, 2]. Epidemiological data also suggest an important impact of NPEV on populations in Africa, Asia, and South America [3–6]. Disease course can range from mild clinical presentations to severe complications such as meningitis, encephalitis, or acute flaccid paralysis [6, 7]. As a result of Enterovirus surveillance programs, and intensified molecular and genetic typing, knowledge on epidemiology and clinical courses of different serotypes and genotypes of NPEV is currently expanding [6]. However, linking genetic variations of E-30 to disease severity or frequency has not been carried out so far. Particularly, the biological mechanisms underlying virulence, fitness, and tropism of different serotypes and genotypes of NPEV are currently undefined, although this knowledge is essential for better control of outbreaks and design of targeted prevention or treatment strategies [6].

Echovirus (E) 30 (E-30), belonging to the genus of enterovirus and a member of the family *Picornaviridae*, is a non-enveloped, single-stranded positive-sense RNA virus with a genome of 7.5 kb. Especially in Europe, E-30 is prevailing in patients with NPEV central nervous system (CNS) infections [8–10]. In parallel, more and more frequent epidemics with E-30 occur in Asia [11] and (South) America [12].

So far, two main routes for NPEV into the immune privileged site of the CNS [13, 14] have been analyzed: passage across the blood-brain barrier (BBB) [15–17] and entry across the blood-cerebrospinal fluid (CSF) barrier (BCSFB) [18–20]. At the level of the BBB, endothelial cells are firmly connected through tight junctions (TJ) and adherens junctions (AJ). In human BBB models applying human brain microvascular endothelial cells (HBMEC) or human cerebral microvascular endothelial cells (hCMEC/D3), it was shown that infection with poliovirus [16], E-6, E-12, and E-30 [17] resulted in damage to the junctional connections, leading to increased paracellular permeability of the barrier. In contrast to the BBB, endothelial cells of the choroid plexus at the level of the BCSFB are fenestrated and the polar choroid plexus epithelial cells, connected through compact TJ and AJ proteins, maintain the highly selective barrier [21, 22]. The effect of different NPEVs on the barrier function and morphology of the BCSFB has not been investigated yet. Previously, it could be demonstrated that E-30 Bastianni can infect human choroid plexus epithelial (HIBCPP) cells and lead to alteration of barrier function [19, 23].

Disruption of the BCSFB and BBB barrier functions may lead to influx of leukocytes into the CNS. In this context, NPEV meningitis is often accompanied by CSF pleocytosis with an early influx of polymorphonuclear (PMN) cells [24–27] followed by increasing numbers of CD4^+^ T-cells [28]. For the BCSFB, it was recently shown that E-30 infection resulted in an enhanced migration of PMN and T-cells in vitro [23]. Leukocyte migration across endothelium or epithelium can occur via two routes, para- and transcellular [29–34]. For transepithelial PMN migration, a dominantly paracellular approach has been observed [35, 36]. Still, it has been noticed that transcellular diapedesis of PMN and T-cells is also a common phenomenon [37–39]. However, investigation of the leukocyte migration pathway in the context of NPEV CNS infection is currently lacking. In this study, we compared the effects of different clinical E-30 outbreak strains (13-311, 13-759, and 14-397) with E-30 Bastianni to obtain information about their in vitro virulence and tropisms within a human BCSFB model. Additionally, the pathway of leukocyte migration following infection with E-30 was analyzed.

## Methods

### Cell lines

Human choroid plexus papilloma (HIBCPP) cells [40] were cultivated on cell culture inserts and were maintained in DMEM/HAM’s F12 1:1 supplemented with 10 or 1% inactivated fetal calf serum (FCS), 4 mM L-Glutamine, streptomycin (100 μg/ml) and penicillin (100 U/ml), insulin (5 μg/ml), previously described by Dinner et al. [41]. For control experiments, the human rhabdomyosarcoma (RD) cell line was used, which is commonly used to propagate NPEV. Culture was performed in DMEM/HAM’s medium containing 10% FCS on 24-well plates.

### Cell culture and barrier integrity evaluation

For the purpose of a filter-based experimental set-up, HIBCPP cells were seeded on inverted culture filter inserts (Millipore and Sarsted, Germany; pore diameter 5.0 μm, pore density 6.0 × 10^5^ pores/cm^2^, area 0.33 cm^2^). The inverted cell culture system was chosen to allow physiologically relevant infection from the basolateral cell side [41, 42]. Evaluation of barrier integrity over the course of the experiment was performed through measurement of transepithelial electrical resistance (TEER) using a tissue voltohmmeter (Millipore, Germany) and permeability analysis of dextran-TexasRed (Invitrogen, Germany) tracer solution (100 μg/ml) following the pre-described methodology [19, 39]. In brief, dextran-TexasRed was added to the upper filter compartment after 24 h of infection for 4 h before its concentration was evaluated using a Tecan infinite M200 Multiwell reader (Tecan, Switzerland). For all experiments, barrier function was monitored through measurement of TEER and flux of TexasRed-labelled dextran over the 28 h.

### Viral propagation, titration, and infection

For this study, four different E-30 strains were used: a clinical isolate, from 1958, E-30 Bastianni (prototype strain) [43], referred to within the figures of the manuscript as E-30 Bast., and three E-30 outbreak strains isolated in Germany 2013 and 2014, referred to as 13-311, 13-759, and 14-397. Virus replication was measured using qPCR analysis as previously described [19]. All strains were provided by the National Reference Centre for Poliomyelitis and Enteroviruses (NRC PE) at the Robert Koch-Institute (RKI) (Berlin, Germany). Selection of inverted filter inserts with HIBCPP cell layers for experiments was based on the TEER reaching appropriate levels from 215 to 675 Ω × cm^2^. All experiments were performed in migration assay medium (MAM), which was not changed over the course of the experiments, and consisted of RPMI-1640 medium supplemented with 5% FCS, HEPES (25 mM), and Glutamine (2%). Infection was performed using a multiplicity of infection (MOI) of 0.7 added to the upper compartment of the filter insert. To identify the virus localization within the in vitro set-up, supernatant from the upper (basolateral cell side) and lower (apical cell side) filter compartment was collected at 0, 5, and 24 h, kept separated, centrifuged at 3500 g for 10 min. Supernatant was stored at − 80 °C. Additionally, filter membranes covered with a HIBCPP cell-layer were cut out using a scalpel and were stored in PBS. Repeated freeze/thaw cycles (2× − 20 °C followed by 1× − 80 °C) followed by centrifugation at 3500 g for 10 min and collection of supernatant was performed. Further analysis via qPCR analysis applying the two-step-RNA-PCR protocol was carried out as described above. For control experiments, RD cells seeded in 48-well plates were infected with different E-30 strains at an MOI of 0.7. Collection of viral particles located in the supernatant or within the cells was performed as described above for HIBCPP cells. TaqMan qPCR analysis (see above) was performed for analysis of virus genome copies.

### Cell viability

HIBCPP cell viability was analyzed through application of the live/dead assay after 28-h infection according to the manufacturer’s instructions (Invitrogen, Germany). In principle, calcein green penetrates the cell membrane and in combination with esterase in the cytoplasm elicits a green fluorescent signal. In contrast, the red fluorescent ethidium homodimer is unable to enter healthy cells; however, penetration and DNA intercalation of damaged cell membranes are possible. The emitted fluorescent signal was analyzed under a fluorescent microscope, and two-dimensional images were recorded. Images were taken with a Zeiss Apotome and ZEN software (Carl Zeiss, Germany) using a ×20/1.4 objective lens. In order to verify virulence of E-30 strains after passage across the HIBCPP cells, infection of RD cell monolayers with the supernatant from uninfected and infected HIBCPP cells was performed. Cytopathic effect was observed via light microscopy.

### Measurement of lactate dehydrogenase (LDH) activity

Cellular integrity up to 28 h post infection was evaluated through measurement of the lactate dehydrogenase concentration in the HIBCPP cell supernatant through application of a commercially available kit (Roche, Germany). Supernatant of uninfected unlysed HIBCPP was used as negative control, and lysed HIBCPP cells (enabling maximum LDH release) were used as a positive control. Both values were used to calculate the percentage of LDH activity after enteroviral infection.

### Immunofluorescence analysis of tight and adherens junction proteins and immune cell migration

In principle, analysis of immunocytochemistry was performed as previously described for primary porcine choroid plexus epithelial cells (PCPEC) and HIBCPP cells [42, 44]. Filter membranes were washed and fixed with 4% formaldehyde (*w*/*v* in PBS) for 10 min at room temperature (RT). Permeability of the membrane was ensured through incubation in 0.5% Triton X-100/ 1% bovine serum albumin (BSA) (*v*/*v* in PBS) for 20 min at RT. Staining was performed for the actin cytoskeleton, TJ proteins ZO1 and occludin, AJ protein E-cadherin, the cell nucleus, and VP1 capsid protein. Immunofluorescent staining was carried out using phalloidin Alexa Fluor 660 (1:60, Molecular Probes, USA) and 4′-6-diamidino-2-phenylindole dihydrochloride (DAPI) (concentration 1:50,000) for 60 min at RT to stain actin and the nucleus, respectively. Primary antibodies, polyclonal rabbit anti-ZO1, anti-occludin, and anti-E-cadherin (Invitrogen, Germany) or monoclonal mouse anti-enterovirus VP1 (Clone 5-D8/1) (Dako, Denmark) were added at a dilution of 1:250 overnight at 4 °C. As secondary antibodies, polyclonal anti-rabbit-IgG Alexa Fluor 488 and 594 were used at a dilution of 1:250 for 1 h at RT. Images were taken with a Zeiss Apotome and Zen software (Carl Zeiss, Germany) using a ×63/1.4 objective lens. For evaluation, Z-stacks of the cell layers were acquired and orthogonal representation enabled immunocytochemical analysis.

For assessment of PMN and T-cell transmigration across the HIBCPP cell layer, an infection experiment over 24 h with an MOI of 0.7 was carried out. As recently described, PMN and naïve CD3^+^ T-cells were isolated from human blood of healthy donors [23]. Each cell population was labelled either with CellTrace™ Calcein red-orange or with CellTrace™ Calcein green (Molecular probes, USA) according to manufacturer’s instructions. After 24 h of infection, 4 × 10^5^ PMN were added into the filter insert. After 2 h of migration, 4 × 10^5^ naïve CD3^+^ T-cells were also added to the filter inserts and both cell populations were incubated for another 2 h (PMN migration 4 h, T-cell migration 2 h). Filters were washed twice in PBS and were fixed with 3.7% formaldehyde for 10 min at RT. The subsequent migration analysis was carried out using immunocytochemical staining of the cell layers as described above.

### Western blot

After 28 h of infection at an MOI of 0.7, HIBCPP cells were washed with PBS. Modified radioimmunoprecipitation assay (RIPA) buffer (containing 50 mM Tris HCL (pH 8), 150 nM NaCl, 0.1% SDS, 1% Triton X-100, 1% natriumdeoxycholate, 1× protease inhibitor cocktail, 1 mM PMSF, 50 mM NaF, 2 mM EDTA, and 1 mM Na3VO4) was used to extract whole protein. The lysates were centrifuged at 18620×*g* for 10 min, and total protein amount was calculated applying the manufacturer’s instructions for the DC Protein Assay according to the Lowry method (BioRad, Germany). A total of 20 μg per sample was mixed with loading buffer and was loaded onto a 4–12% Bis-tris gel (Invitrogen, Germany) followed by overnight blot onto a nitrocellulose membrane using standard conditions. Primary antibodies used for the immunoblotting were rabbit-anti-ZO1, rabbit-anti-occludin, rabbit-anti-E-cadherin (Cell Signaling, UK, all 1:500 dilution), and mouse-anti-β-actin (Sigma-Aldrich, Germany; 1:10,000 dilution). Secondary antibodies used for detection were either anti-rabbit or anti-mouse horseradish peroxidase (HRP)-conjugated (Millipore, USA; 1:5000 dilution). Visualization was ensured using the respective substrate from Immobilon Western Kit (Millipore, USA).

### Transmission electron microscopy

To further analyze PMN and T-cells migrating across the HIBCPP cells, we prepared filter membranes from different experimental conditions for transmission electron microscopy (HIBCPP + PMN + T-cells ± chemokine ± E-30 Bastianni) as described previously [39]. Briefly, after 28 h of infection and subsequent leukocyte migration, the filter inserts were fixed over night at 4 °C in 2% glutaraldehyde/0.1 M cacodylate buffer (pH 7.4) embedded in Araldite (Science Services, Germany) and ultrathin sections were performed using an ultramicrotome (Ultracut R) (Leica, Germany). Samples were analyzed with a Zeiss Em 10 Transmission electron microscope (Zeiss, Germany).

### Focused ion beam/scanning electron microscopy (FIB/SEM)

For scanning electron microscopy (SEM), gold palladium sputter-coated membranes were attached on specific SEM sample holders. Regions of interest for three-dimensional imaging were decided upon after light microscope analysis of thin cut membrane slices. Serial sectioning imaging was performed with a Zeiss Crossbeam 550 gallium FIB-SEM instrument (Zeiss, Germany) operating at low voltage (SEM EHT = 1.7 kV) [45, 46]. FIB slices were prepared, and images acquired at variable regions of interest using the ATLAS 3D nanotomography package (Zeiss, Germany) and an energy-selective in-lens backscattered electron detector (EsB) with voxel resolutions of 10 × 10 × 10 nm (sample HIBCPP + PMN + T-cells + E-30 + CXCL12) and 8 × 8 × 8 nm (sample HIBCPP + PMN + T-cells + E-30 + IL8). Two-dimensional image filtering of the raw data (non-local means: both samples; Gaussian: sample HIBCPP+PMN + T-cells+E-30 + CXCL12) and three-dimensional reconstruction and video visualization were performed without additional slice registration using the commercial software package ORS Visual SI [47].

### Quantification of viral genome copies via qPCR

Quantification of viral genome copies was performed on total RNA extracted from the supernatant and cell lysate collected throughout the infection course. Extraction was automatically performed using the EZ1 Virus Mini Kit (Qiagen®, Germany) following the manufacturer’s instructions. Quantitative real-time PCR (qPCR) analysis was carried out using quantitative two-step TaqMan qPCR. Primers targeting the 5′UTR region were Ent411F-5′-GACATGGTGCTGAAGAGTCTATTGA-3′ and Ent486R-5′-GCTCCGCAGTTAGGATTAGCC-3′. The probe used was Ent443T-5′FAM-GTAGTCCTCCGGCCCCTGAATGC-TAMRA-3′. Reverse transcription was performed with Ent486R using Sensicript RT (Qiagen, Germany) according to manufacturer’s recommendations. Standard protocols and conditions were applied during amplification using a 2× universal-master-mix (Applied Biosystems, Germany) on an ABI 7500 thermal cycler (Applied Biosystems, Germany). As standards, plasmids that encompass the amplified region were created and serially diluted after purification. Standard graphs of the C_*T*_ values obtained from serial dilutions of the standards (5000, 500, and 50 copies) were constructed and the numbers of specific genomes were calculated by the software. This method was used to analyze the number of viral genome copies, without categorizing in infectious virus and non-encapsidated viral RNA.

### Quantification of viral infection via immunofluorescence

HIBCPP cell infection at an MOI of 0.7 with each strain was performed for 28 h and subsequently the filters were washed with PBS and stained. The immunofluorescent staining for DAPI (HIBCPP nucleus) and viral capsid protein (VP) 1 was performed as described in the “Immunofluorescence analysis of tight and adherens junction proteins and immune cell migration” section of “Methods.” Infected cells were identified through the presence of intracellular VP1 staining and compared to the total amount of HIBCPP cells growing on the filter. Percentage of E-30-infected HIBCPP cells was calculated by counting 10 fields of view (142.1 μm × 106.48 μm) per filter in four independent experiments. The overall infection capabilities were calculated using the average amount of infected cells per field and multiplied with an area coefficient. The size of the filter membranes was 0.33 cm^2^.

### Complete genomic sequencing

The RNA was extracted from 140 μl of the supernatant of virus-infected cell cultures using the QIAamp Viral RNA Mini Kit (Qiagen, Germany) according to the manufacturer’s instructions. Synthesis of the cDNAs from purified RNAs was performed using SuperScript™ III Reverse Transcriptase (Invitrogen, Germany) according to the manufacturer’s instructions. Primers used for amplification and sequencing were designed according to conserved sequences in the whole genome sequences reported in Genbank for Echovirus 30 and are available on request. The amplification was performed by using the Phusion® High-Fidelity PCR Master Mix with HF Buffer (New England Biolabs, USA). The 1–4 kbp PCR products obtained were purified using the Gel Extraction Kit (Qiagen, Germany). The nucleotide sequences were determined at GATC (Biotech AG, Germany) with an ABI 3730xl DNA analyzer. Forward and reverse sequencing reactions were carried out with the oligonucleotides used in gene-amplification reactions. The sequences were deposited in the GenBank database under accession number KY888272-KY888275.

### Phylogenetic analysis of echovirus 30 nucleotide sequences

Nucleotide regions encoding for the capsid proteins (P1 region) were used as a query sequence in a BLAST search against the Genbank non-redundant database. Prototype strains of Echovirus 11 (X80059.1) and Echovirus 6 (AY3025588.1) were used as an outgroup. The sequences were aligned using ClustalW as implemented in MEGA 7.0. A phylogenetic tree was calculated with the neighbor-joining method using the Tamura-Nei’s model for nucleotide substitution. The reliability of the phylogenetic topologies was determined by bootstrap-resampling test with 1000 replicates.

### Statistics

Statistical analysis was performed using the SAS system, release 9.3 (SAS Institute Inc., Cary, USA). Virus strains (E-30 Bastianni, 13-311, 13-759, and 14-397) and time points were “fixed factors” and the day of the experiment “random factor” in the SAS procedure SAS MIXED which was applied to calculate significance in dextran flux and TEER. For viral load distribution a “Poisson-Regression” using the “Tukey–Kramer” comparison calculation was applied. If not otherwise stated, result values are given as mean + SD and the level of significance is denoted in each figure using either * or #.

## Results

### Alteration of barrier function of HIBCPP cells after infection with E-30 is strain-dependent

**Fig. 1:**
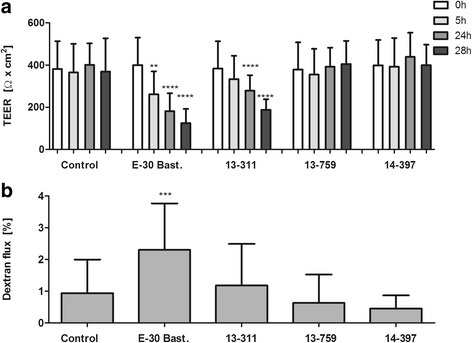
E-30 strain-specific effects on barrier integrity of HIBCPP cells Barrier integrity of HIBCPP cells was evaluated via measurement of the transepithelial electrical resistance (TEER) (**a**) and the paracellular permeability for the low molecular weight molecule dextran-TexasRed (3000 kDa) (**b**) at indicated time points after infection with E-30 Bastianni*,* 13-311, 13-759, or 14-397 and compared to uninfected controls (**a**) TEER values at the start of the experiment (white bars), after 5 h (light gray), after 24 h (medium gray) and after 28 h (dark gray) are shown. Data are shown as mean + SD of 11 independent experiments carried out in triplicates. *p* values compare the TEER at 5, 24, and 28 h after infection for each viral strain with the respective control at 0 h (**p < 0.01, ****p < 0.0001). **b** Evaluation of the paracellular permeability was carried out after 24 h of infection of HIBCPP cell layers with different viral strains via the addition of dextran to the upper compartment of the filter for 4 h. Data shown are mean + SD of five independent experiments each performed in triplicates. *p* values were calculated when comparing paracellular permeability of all infected and uninfected conditions to E-30 Bastianni infected HIBCPP cells (***p < 0.001)

To evaluate the effects of infection with different E-30 outbreak strains at an MOI of 0.7 over 28 h on the barrier integrity of HIBCPP cells, TEER, and the paracellular permeability for dextran were measured at indicated time points. Over the time course of the experiment, the TEER values of the uninfected controls showed no significant changes (Fig. 1a). A similar pattern and also no significant changes were observed upon infection of HIBCPP cells with the strains 13-759 and 14-397 (Fig. 1a). In contrast, both E-30 Bastianni and 13-311 triggered a significant decrease in TEER after 5, 24, and 28 h of infection compared to respective TEER values measured at the start of the experiment (Fig. 1a). Interestingly, the dextran flux evaluation (Fig. 1b) showed that E-30 Bastianni was the only strain which caused a significant increase in dextran flux with 2.30 ± 1.46% diffusion across the HIBCPP layer compared to the uninfected control (*p* = 0.0020). Strains 13-311 (1.18 ± 1.31%), 13-759 (0.63 ± 0.90%), and 14-397 (0.45 ± 0.42%) did not cause a significant increase in paracellular permeability and the values remained comparable to the uninfected control (0.94 ± 1.06%) (Fig. 1b).

### E-30 strain-dependent alterations of the morphology of tight and adherens junctions

**Fig. 2:**
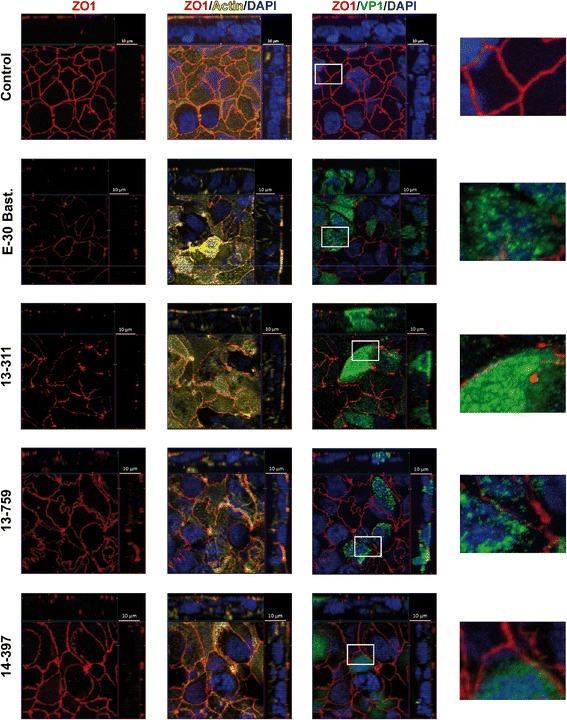
E-30 infection of HIBCPP cells leads to strain-dependent alteration of the TJ morphology involving ZO1. HIBCPP cells were infected with E-30 Bastianni*,* 13-311, 13-759, and 14-397 for 28 h and ZO1 staining was compared to uninfected controls. Cell layers were stained for nuclei with 4, 6-diamidino-2-phenylindole (DAPI) (shown here in blue), actin cytoskeleton with Phalloidin (shown here in yellow), the viral capsid protein 1 (VP1) (shown here in green) and ZO1 (red). Z-stack images were taken from regions with infected cells using an Apotome and represented through display of a cross-section through the z-plane of multiple slices in combination with a two-dimensional view from the top of the cell layer. For each strain we included a 400× enlarged image of an area which we considered a representative frame. The polarized cells are oriented with the apical side pointing up or to the right and basolateral downwards or to the left. Three images per strain showing different grouping of parallel staining in combination with a zoomed in view are displayed horizontally (column one: only ZO1; column two: DAPI, actin, and ZO1; column three: DAPI, VP1, and ZO1); E-30 strains are listed vertically. The images shown are representative examples of multiple stainings taken from six independent experiments each performed in duplicates

**Fig. 3:**
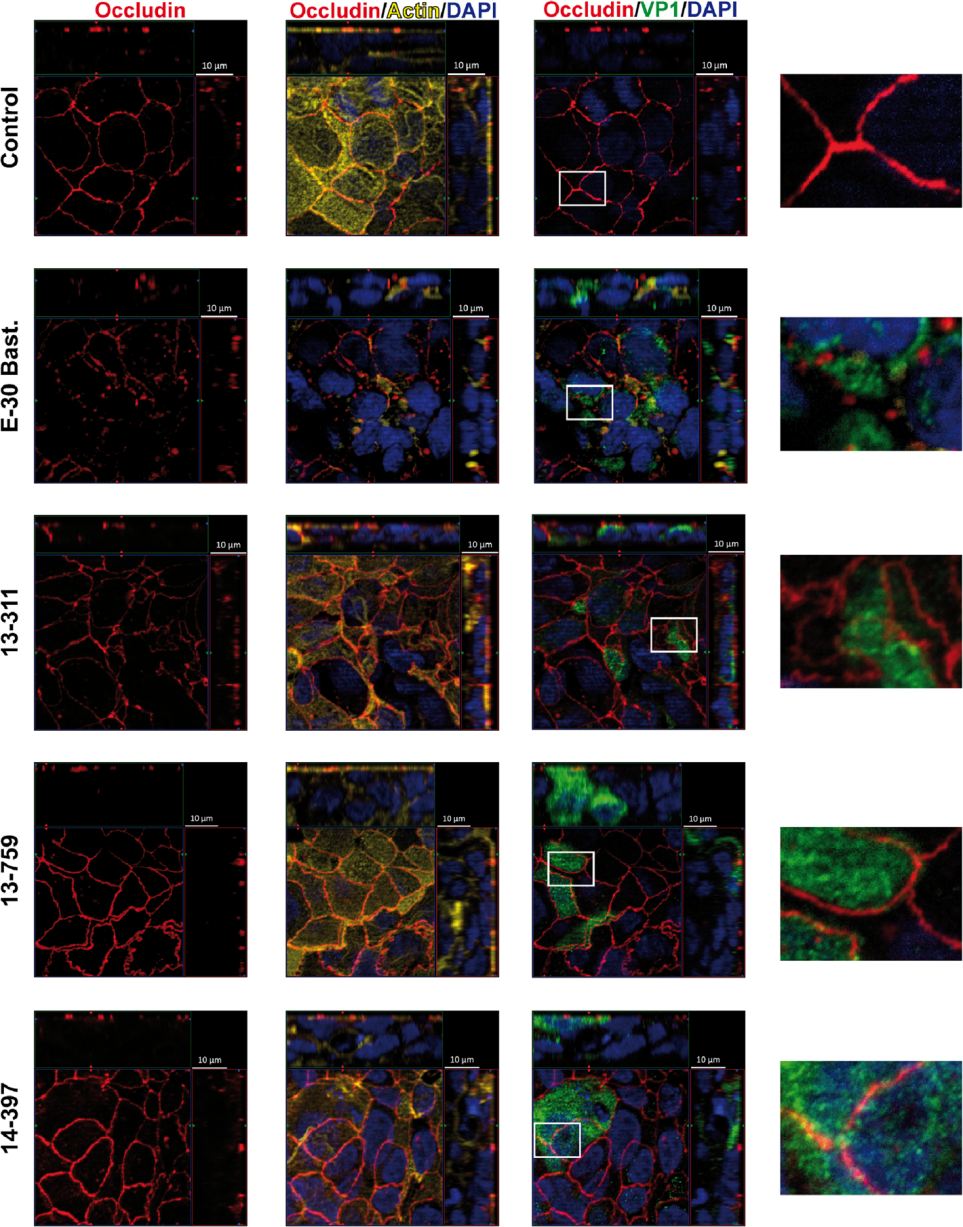
E-30 infection of HIBCPP cells leads to strain-dependent alteration of TJ morphology involving occludin. HIBCPP cells were treated analogous to procedures described for Fig. 2. Cell layers were stained for nuclei with DAPI (shown here in blue), actin cytoskeleton with phalloidin (shown here in yellow), VP1 (shown here in green) and occludin (red). For detailed description of image acquisition and preparation please refer to Fig. 2. Three images per strain showing different grouping of parallel staining in combination with a zoomed in view are displayed horizontally (column one: only occludin; column two: DAPI, actin, and occludin; column three: DAPI, VP1, and occludin); E-30 strains are listed vertically. The images shown are representative examples of multiple stainings taken from six independent experiments each performed in duplicates

**Fig. 4:**
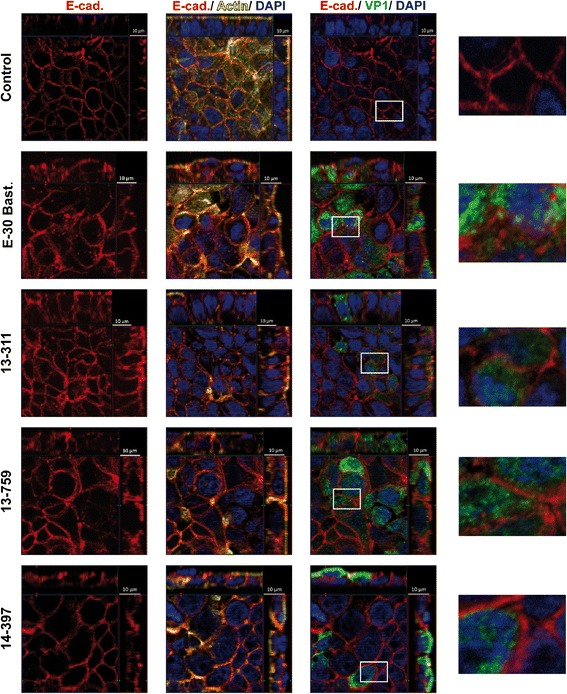
E-30 infection of HIBCPP cells leads to strain-dependent alteration of AJ morphology involving E-cadherin. HIBCPP cells were treated analogous to procedures described for Fig. 2. Cell layers were stained for nuclei with DAPI (shown here in blue), actin cytoskeleton with phalloidin (shown here in yellow), VP1 (shown here in green), and E-cadherin (red). For detailed description of image acquisition and preparation, please refer to Fig. 2. Three images per strain showing different groupings of parallel staining in combination with a zoomed in view are displayed horizontally (column one: only E-cadherin; column two: DAPI, actin, and E-cadherin; column three: DAPI, VP1, and E-cadherin); infection strains are listed vertically. The images shown are representative examples of multiple stainings taken from six independent experiments each performed in duplicates

As the barrier function of HIBCPP cells is mainly established via a complex network of TJs similar to other polarized epithelial cells [42, 48], we were interested in the morphology of TJ and AJ following infection with the different E-30 strains, which we analyzed via immunofluorescence analysis of ZO1, occludin, and E-cadherin (Figs. 2, 3, and 4). In the uninfected controls, a continuous “puzzle-like” staining of ZO1 was detectable (Fig. 2). It is expected localization, at the apical side of the cell, was verified with the orthoscopic view. Infection with E-30 Bastianni or 13-311 led to extensive disruption of ZO1 of infected neighboring HIBCPP cells (Fig. 2). The ZO1 of uninfected HIBCPP cells within the same layer of cells was not affected. Similar effects were observed for occludin after infection of HIBCPP cells with E-30 Bastianni or 13-311 (Fig. 3). In contrast, the strains 13-759 and 14-397 had no apparent effect on ZO1 and occludin topology (Figs. 2 and 3). At the level of AJs, both the infection with E-30 Bastianni and to a much lesser extent with 13-311 led to alterations of the morphology (Fig. 4). However, the strains 13-759 and 14-397 had no effect on E-cadherin morphology (Fig. 4). Actin staining remained unaffected under any condition (Figs. 2, 3, and 4; column 2).

To exclude that differences in cytotoxicity of the different E-30 strains were responsible for the observed effects on the morphology of TJ and AJ, we tested both the viability and release of LDH from HIBCPP cells upon infection with E-30. Analysis of viability after 28 h of infection showed no significant increase in dead cells for any of the viral strains compared to the uninfected control (Additional file 1A). As a second parameter, we measured LDH release. These measurements indicated only minor effects of all E-30 strains on HIBCPP cells when compared to uninfected controls, but no significant strain-dependent differences (Additional file 1B).

### Junctional protein expression in HIBCPP after E-30 infection is not altered

**Fig. 5:**
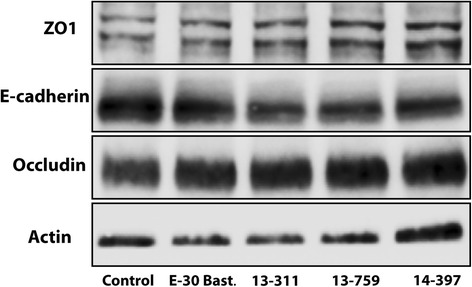
E-30 infection of HIBCPP cells does not affect tight and adherens junction whole protein expression. Western blot of total protein lysate of HIBCPP cells after 28 h of infection with E-30 Bastianni, 13-311, 13-759, and 14-397 and of uninfected control samples. Antibodies were directed against ZO1 (220 kDa), E-cadherin (120 kDa), occludin (65 kDa), and actin (42 kDa). Experiments were carried out in triplicates, and a representative blot was used for illustration taken from of six independent experiments

As we observed, strain-specific alterations to the morphology of TJ and AJ of the infected HIBCPP cells were investigated, whether protein expression was affected in a comparable manner. These findings indicate that protein expression of the TJ proteins ZO1 (220 kDa) and occludin (65 kDa) as well as of the AJ protein E-cadherin (120 kDa) was not considerably altered (Fig. 5).

### Infection with E-30 promotes paracellular and transcellular PMN and T-cell transmigration

**Fig. 6:**
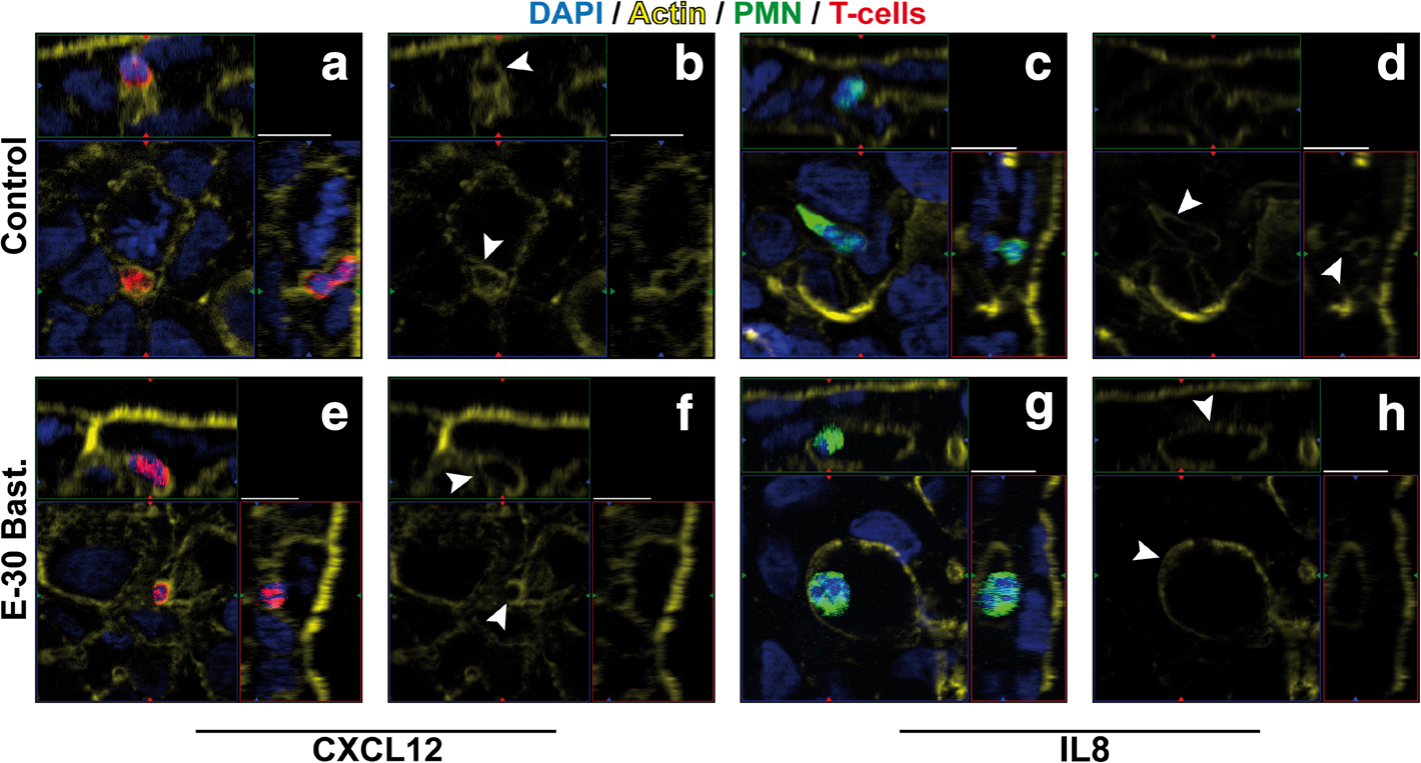
Immunofluorescence analysis of leukocyte migration pathway across the HIBCPP. Immune cell migration across HIBCPP monolayers infected with E-30 Bastianni was analyzed using immunofluorescent staining. Infection for 28 h with E-30 Bastianni was carried out before staining the filters for nuclei with DAPI (shown here in blue), actin skeleton with phalloidin (shown here in yellow), PMN (shown here in green), and T-cells (shown here in red). For detailed description of image acquisition and preparation, please refer to Fig. 2. Image pairs show regions of interest and a single channel view of actin expression. White arrowheads indicate the localization of the leukocyte in relation to the actin skeleton. The surrounding of leukocytes by intercellular actin borders which are connected to the apical epithelial cell membrane is in favor of paracellular diapedesis (**b**, **d**, **f**), whereas the localization of the leukocyte in clear distance of the actin skeleton is in favor of transcellular diapedesis (**h**). Images were taken for each of the following conditions: HIBCPP cells + PMN and T-cells + CXCL12 = **a**, **b**; HIBCPP cells + PMN and T-cells + E-30 Bastianni + CXCL12 = **e**, **f**; HIBCPP cells + PMN and T-cells + IL8 = **c**, **d**; HIBCPP cells + PMN and T-cells + E-30 Bastianni + IL8 = **g**, **h**. These are representative images of three independent experiments, each carried out in duplicates and analyzed with a minimum of four z-stacks per filter

Based on previous observations at the BBB and BCSFB, we hypothesized that the preferred migration pathway of leukocytes during sequential transmigration into the CNS may be dependent on the level of barrier impairment. Therefore, we carried out extensive analyses, applying IF staining, TEM and FIB/SEM, and captured PMN and T-cells during their passage across the HIBCPP layer. When analyzing images from IF, the location of the immune cell actin skeleton in relation to the actin cytoskeleton of the HIBCPP cells indicates both para- and transcellular transmigration with or without E-30 infection (Fig. 6). The surrounding of leukocytes by intercellular actin borders which are connected to the apical epithelial cell membrane is in favor of paracellular diapedesis (white arrowheads in Fig. 6b–f). We could also visualize an elongated PMN body squeezing through the cell borders of HIBCPP cells indicating paracellular diapedesis (Fig. 6c, d). In contrast, when the migrating immune cell is localized in clear distance to the actin cytoskeleton of the HIBCPP cells, transcellular diapedesis is more likely. As shown in Fig. 6g, h, in an experiment involving infection with E-30 Bastianni, the body of a transmigrating PMN is in clear distance to the HIBCPP cell membrane indicating transcellular PMN migration.

**Fig. 7:**
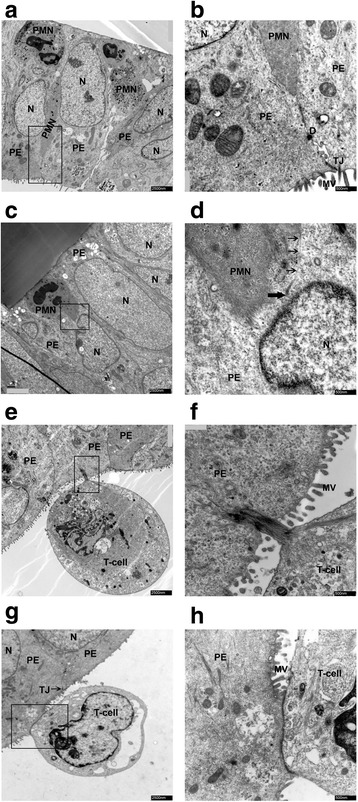
Transmission electron microscopic analysis of migration pathways of immune cells across the HIBCPP cells. Migration pathways of PMNs and naive T-cells across HIBCPP cells were identified through electron microscopy. Images were taken for each of the following conditions: HIBCPP cells + PMN + T-cells + CXCL12 = **a**, **b**; HIBCPP cells + PMN + T-cells + CXCL12 + E-30 Bastianni = **c**, **d**, and **g**, **h**. HIBCPP cells + PMN + T-cells + IL8 + E-30 Bastianni = **e**, **f**; The horizontal image pairs are an overview and an enlarged frame of the HIBCPP cell layer with migrating leukocytes. (**a**, **b**) Paracellular migration of PMN can be identified, as the elongated PMN is squeezing through tight epithelial cells. (**c**, **d**) Transcellular migration of PMN, entering the cell layer from the basolateral side; single arrow = cell membrane; thick arrow = end of membrane. (**e**, **f**) Transcellular T-cell migration can be seen, as the leukocyte is leaving the cell layer towards the apical HIBCPP cell side. Cellular connection through actin filament is in clear distance from intact TJ (**g**, **h**). Transcellular T-cell migration is visible and confirmed through the connection between the T-cell and the HIBCPP cell at clear distance from intact TJ. These are representative images of two independent experiments, each carried out in triplicates. Scale bar A, C, E, and I: 2500 nm, B, D, F and H: 500 nm. N = Plexus epithelial cell nucleus; PE = plexus epithelial cell, D = desmosome, MV = microvilli, TJ = tight junctions, PMN = polymorphonuclear neutrophil

In order to verify the observations made during IF staining analysis, we further carried out a thorough electron microscopic analysis. Here, we could confirm the IF analysis (Fig. 7). In Fig. 7a–b paracellular, in Fig. 7c–h transcellular migrations of leukocytes are shown. Paracellular PMN migration could be confirmed through the, often elongated, PMN pushing between the cell borders of neighboring epithelial cells, migrating from the basolateral cell side towards the apical lumen (Fig. 7a). In the enlargement, the PMN is visible directly in front of the intact cellular junction complex (Fig. 7b). Besides, undergoing paracellular transmigration PMN were also observed to migrate via the transcellular route (Fig. 7c, d). The PMN cell membrane (small arrows) and extensions in the direction of the migration process (thick arrow) were observed (Fig. 7d). A T-cell that is just about to exit the HIBCPP cell on its apical side, whilst still connected through dense actin filament to the plexus epithelial cell, demonstrates the transcellular route (Fig. 7e, f). Finally, a most likely transcellular migrating T-cell is shown that is leaving the monolayer, but is still connected with HIBCPP cell over a larger area of the apical membrane (Fig. 7g, h).

**Fig. 8:**
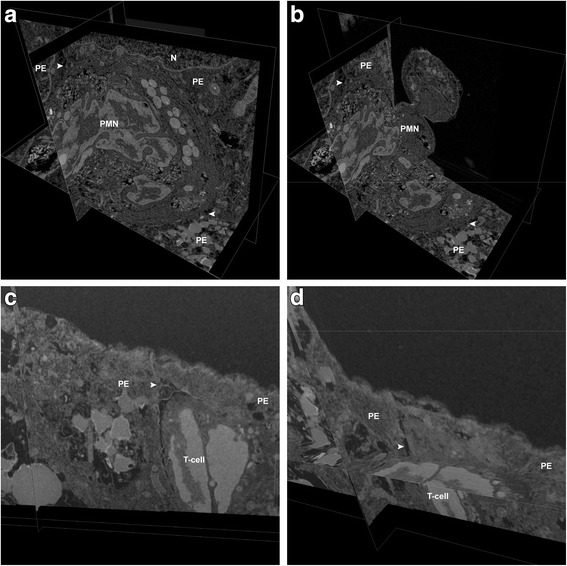
Focal ion beam microscopy analysis of migration pathway of PMN and T-cells cells across the HIBCPP cells. Further analysis of the migration pathway of PMN and T-cells over uninfected and infected HIBCPP cell layers. Shown here are 3D images from HIBCPP + E-30 Bastianni + PMN + T-cells + IL8 (**a**, **b**) and HIBCPP + E-30 Bastianni + PMN + T-cells + CXCL12 (**c**, **d**). The first pair of images (**a**, **b**) shows a PMN moving paracellularly between choroid plexus epithelial cells (PE) and was taken from freeze-frames from Additional file 5. Clear intercellular gaps are visible around the periphery of the PMN and especially at the tricellular borders (arrowheads). The PMN is moving through the pore, into the HIBCPP layer, and in between HIBCPP cells. **c**, **d** Illustrate a transcellular migration of a T-cell across a HIBCPP cell with clear distance to any cell junction complex. These images were taken from freeze-frames from Additional file 8. Labelled are N = epithelial cell nucleus; PE = plexus epithelial cell, PMN = polymorphonuclear neutrophil

For leukocyte migration pattern analysis across cellular layers a three-dimensional reconstruction of the migrating cell and its surrounding is of great advantage. We therefore performed FIB/SEM tomography of the HIBCPP layer with migrating leukocytes to further characterize the migration pathways. We could again demonstrate the paracellular migration of a PMN between two adjacent HIBCPP cells (Fig. 8a, b; Additional files 2, 3, 4, and 5; condition HIBCPP+PMN + T-cells+E-30 + IL8). The PMN is just about to enter the cell layer from the basolateral side and two separate membranes of the PMN and the adjacent HIBCPP cells are clearly observed (Fig. 8a, b; arrowheads). Thus, we could identify cellular borders and intercellular gaps, which are formed during paracellular movement of the PMN (arrowheads). When adding all slices into the three-dimensional construct, we could identify that the visible PMN is entering the HIBCPP layer from the basolateral side through the filter pore (Additional files 2, 3, 4, and 5). The PMN squeezes through the pores of the membrane moving from the basolateral HIBCPP cell side towards the apical cell side. In the flight mode of Additional file 4, the probe is demonstrated as a cube and sliced along the *x*- and *z*-axis. In the Additional file 5 the non-local means projection is visualized. We further analyzed a second stimulation condition (HIBCPP + PMN + T-cells + E-30 + CXCL12), where the transcellular migration of a T-cell is displayed (Fig. 8c, d, Additional files 6, 7, and 8). The T-cell paves its way from the basolateral to the apical cell side (arrowheads). The video of Additional file 6 shows serial images of the raw data. Additional file 7 shows the same data with inverted grey-level distribution. Here, the clear distance to the HIBCPP cell borders is demonstrated and that the T-cell lies within the HIBCPP cell body. Lastly, in the Additional file 8 shows projections of the three-dimensional volume (after two-dimensional Gaussian and non-local means filtering of the raw data).Additional file 2: Paracellular PMN migration across HIBCPP in FIB/SEM–raw data. FIB/SEM serial sections of paracellular polymorphonuclear neutrophil (PMN) migration through HIBCPP cells. Shows raw data SEM images from the condition HIBCPP+PMN + T-cells+E-30 + IL8. The video shows a paracellular migrating PMN presented in Fig. 8a, b in orthoslices. (AVI 12267 kb).Additional file 4: Paracellular PMN migration across HIBCPP in FIB/SEM–3D slices. FIB/SEM serial sections of paracellular polymorphonuclear neutrophil (PMN) migration through HIBCPP cells. Shows SEM images taken from the condition HIBCPP+PMN + T-cells+E-30 + IL8, visualized as a three-dimensional volume and sliced along the *x*- and *z*-axis. The video shows a paracellular migrating PMN presented in Fig. 8a, b in orthoslices. (AVI 15748 kb)Additional file 5: Paracellular PMN migration across HIBCPP in FIB/SEM–NLM projection. FIB/SEM serial sections of paracellular polymorphonuclear neutrophil (PMN) migration through HIBCPP cells. Shows SEM images from the condition HIBCPP+PMN + T-cells+E-30 + IL8, visualized as a three-dimensional volume at different projections. The video shows a paracellular migrating PMN presented in Fig. 8a, b in orthoslices. (AVI 19100 kb)Additional file 6: Transcellular T-cell migration across HIBCPP in FIB/SEM–raw data. FIB/SEM serial sections of transcellular T-cell migration through HIBCPP cells. Shows raw data SEM images from the condition HIBCPP + PMN + T-cells+E-30 + CXCL12. The video shows a transcellular migrating T-cell presented in Fig. 8c, d in orthoslices. (AVI 8145 kb)Additional file 7: Transcellular T-cell migration across HIBCPP in FIB/SEM-inverted images FIB/SEM serial sections of transcellular T-cell migration through HIBCPP cells. Shows inverted SEM images from the condition HIBCPP+PMN + T-cells+E-30 + CXCL12. The video shows a transcellular migrating T-cell presented in Fig. 8c, d in orthoslices. (AVI 12000 kb)Additional file 8: Transcellular T-cell migration across HIBCPP in FIB/SEM–NLMG projection. FIB/SEM serial sections of transcellular T-cell migration through HIBCPP cells. Shows SEM images from the condition HIBCPP+PMN + T-cells+E-30 + CXCL12, visualized as a three-dimensional volume at different projections. The video shows a transcellular migrating T-cell presented in Fig. 8c, d in orthoslices. (AVI 11252 kb)

### Distinct E-30 strain-dependent replication efficacy in HIBCPP and RD cells

**Fig. 9:**
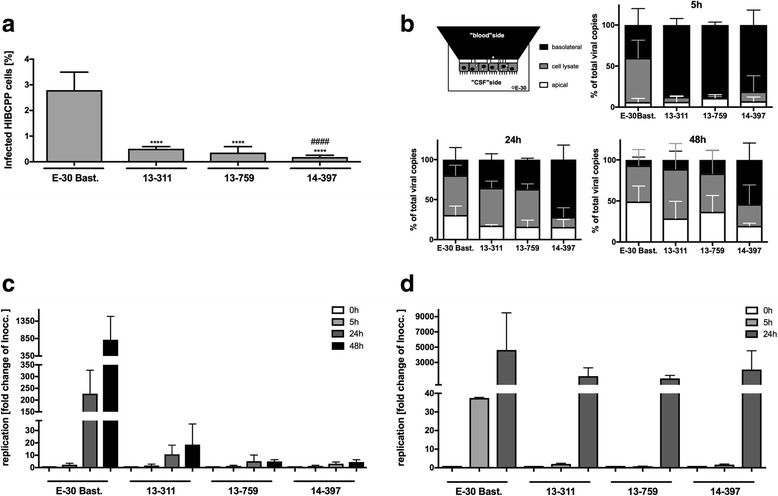
E-30 strain-specific variance in replication and viral dissemination in HIBCPP and RD cells. HIBCPP cells or RD cells were infected with E-30 Bastianni*,* 13-311, 13-759, and 14-397 for up to 48 h (MOI 0.7). **a** Firstly, filters were stained for DAPI and VP1. Then quantification of infected cells was carried out following the procedure described in the material and methods section. Bars display the percentage of infected HIBCPP cells. Data are shown as mean + SD from three independent experiments carried out in duplicates. *p* values were calculated for the comparison of E-30 Bastianni-infected HIBCPP cells against infected with either outbreak strain (****p < 0.0001) or for comparison of 13-759 and 14-397 with 13-311 (####*p* < 0.0001). **b** The distribution of viral genomes was analyzed by qPCR within the cells and two extra-cellular sides. At each respective time point (5, 24, and 48 h), supernatant from both the filter compartment (basolateral cell side) and the well compartment (apical cell side) was collected. Additionally, filter membranes covered with a HIBCPP cell-layer were lysed to assess cell-associated virus. The upper left shows a schematic view of the different compartments compared in this experiment: the basolateral compartment (black), the cell lysate (gray), and the apical compartment (white). Basolaterally (shown here in black), cell associated (shown here in gray), and apically (shown here in white) located viral particles are represented as percentage of total viral particles for the particular strain. On the upper right the results after 5 h of infection, on the lower left the results after 24 h of infection, and on the right the results after 48 h of infection are shown. Data shown are mean + SD from at least three independent experiments carried out in triplicates. **c** After infection of HIBCPP cells, collection of cell free (from the filter and well compartments) and cell-associated virus was performed at indicated time points: 0 h (white bars), 5 h (light gray bars), 24 h (dark gray bars), and 48 h (black bars). The measurement of viral genome copies was carried out with qPCR. Data are shown as mean + SD for three independent experiments performed in duplicates (*p < 0.05, ****p < 0.00001). **d** After infection of RD cells, collection of cell free (from the well) and cell-associated virus particles was carried out at indicated time points: 0 h (white bars), 5 h (light gray bars), and 24 h (dark gray bars). Data are shown as mean + SD taken from four independent experiments carried out in triplicates

To explore possible reasons for the strain-dependent observed effects on barrier function and morphology, we first focused on calculating the amount of infected HIBCPP cells after infection with each strain. After 28 h, significantly more cells were infected by E-30 Bastianni (2.7940 ± 0.7012% *p* < 0.0001) compared to the outbreak strains. In addition, strain 13-311 (0.5071 ± 0.0858%) infected significantly more HIBCPP cells than strain 14-397 (0.1836 ± 0.0713% *p* < 0.0001)]. There was no significant difference between strain 13-311 (0.5071 ± 0.0858%) and strain 13-759 (0.3544 ± 0.2335%) (Fig. 9a).

To further characterize the viral strains, we monitored the distribution of viral genomes within our in vitro system and determined the percentage of cell-associated virus or viral genomes recovered from the basolateral or apical HIBCPP cell supernatant. After 5 h exclusively for E-30 Bastianni already 33 ± 18% of the total viral genomes were found within the HIBCPP cells. For the outbreak strains most of the viral genomes were still located in the basolateral filter compartment (Fig. 9b). After 24 h for E-30 Bastianni 31 ± 11% of viral genomes were found in the apical and 20 ± 15% were detected in the basolateral filter compartment. In comparison, for strains 13-311, 13-759, and 14-397, 17 ± 1%, 16 ± 8%, and 16 ± 9%, respectively, were detectable within the apical filter compartment at this time point. Strain 13-311 and 13-759 showed similar distribution with 35 ± 7% and 37 ± 2% found in the basolateral supernatant and 47 ± 7% and 47 ± 6% cell-associated viral genomes. Noteworthy, strain 14-397 behaved different to the other strains with only 12 ± 12% of the viral genomes located within the cells, but a majority of viral genomes 72 ± 18% were still detected basolaterally (Fig. 9b). Moreover, in contrast to the other outbreak strains, the pattern of the distribution of viral genomes of strain 14-397 does not vary between 5 and 24 h of infection. The prolongation of the infection period to 48 h does not change the pattern of effects on barrier integrity and viral particle distribution of the different strains on HIBCPP cells (Additional file 9). Although 13-759 and 13-311 are comparable concerning viral particle distribution and replication rates (Fig. 9b, c), they show opposing effects on barrier integrity (Additional file 9). 14-397 is not catching up in regard of replication rate (Additional file 9). Interestingly, for all E-30 strains, their progeny harvested at the apical side of the cellular barrier was infectious (Additional file 10). Still, the strain E-30 Bast. was causing the strongest cytopathic effect on RD cells, whereas the outbreak strains showed less (13-311> > 13-759 > 14-397) (Additional file 10).

In HIBCPP cells, in contrast to strong viral replication rates measured via qPCR as viral genome copies/ml for E-30 Bastianni, all three outbreak strains showed lower replication rates in a comparable range (Fig. 9c). Still, there was a trend for the 14-397 strain showing the lowest replication rate over the time course of the experiment (Fig. 9c). To investigate whether the differences in viral replication cycles between E-30 Bastianni and the outbreak strains were unique for HIBCPP cells, we carried out infection experiments with the outbreak strains in RD cells (Fig. 9d). Interestingly, the time kinetics of viral replication in RD cells differed. In both cell types, E-30 Bastianni showed the fastest replication cycles, multiplying within 24 h from 2.74 × 10^5^ ± 9.95 × 10^4^ to 4.85 × 10^7^ ± 2.46 × 10^7^ viral copies/ml in HIBCPP cells and from 3.43 × 10^4^ ± 3.97 × 10^2^ to 1.60 × 10^8^ ± 1.69 × 10^8^ in RD cells. Overall, in contrast to the behavior in HIBCPP cells, the outbreak strains do not show defects in the viral replication cycles in RD cells (Fig. 9c, d). The pattern of viral replication of the different outbreak strains after 5 h was comparable to the pattern observed for HIBCPP cells at 24 h. Still, after 24 h of infection, the three outbreak strains reached comparable levels of viral replication in RD cells (Fig. 9c, d).

### Outbreak strains differ in nucleotide and amino acid sequences

Genetic evolution of viral strains may lead to important variation in their genotypes, which can influence their phenotypes, i.e., viral replication within human host cells [49]. Consequently, we sequenced, aligned, and compared the genomes of all used strains (Additional files 11 and 12). The full genomes showed 81.1–81.6% nucleotide (nt) and 96.0–96.4% amino acid (aa) identities with E-30 Bastianni, and 83.4–99.1% nt and 96.6–99.5% aa similarity with each other. Alignment of polyprotein sequences identified several clusters of the frequent aa variations between the isolates and E-30 Bastianni. These clusters were mostly located at 15-24, 573-655, 690-725, 831-886, and 1043-1157 aa positions of the P1 and P2 regions (Additional files 11 and 12). Among these were eight mutations that located in the solvent-exposed areas of the VP1 protein [50]. Additional point variations were equally allocated all over the polyprotein sequences. Interestingly, only 10 aa differences were found between the polyprotein sequences of the isolates 13-311 and 14-397: T15S, S47T, E227G, T463A, A513V, A578T, P826Q, L1104P, V1801I, and V2121I (Additional file 11). Contrary, the 13-759 strain was markedly different displaying 70 and 74 aa differences, when compared to the isolates 13-311 (Additional files 11 and 12) and 14-397, respectively.

**Fig. 10:**
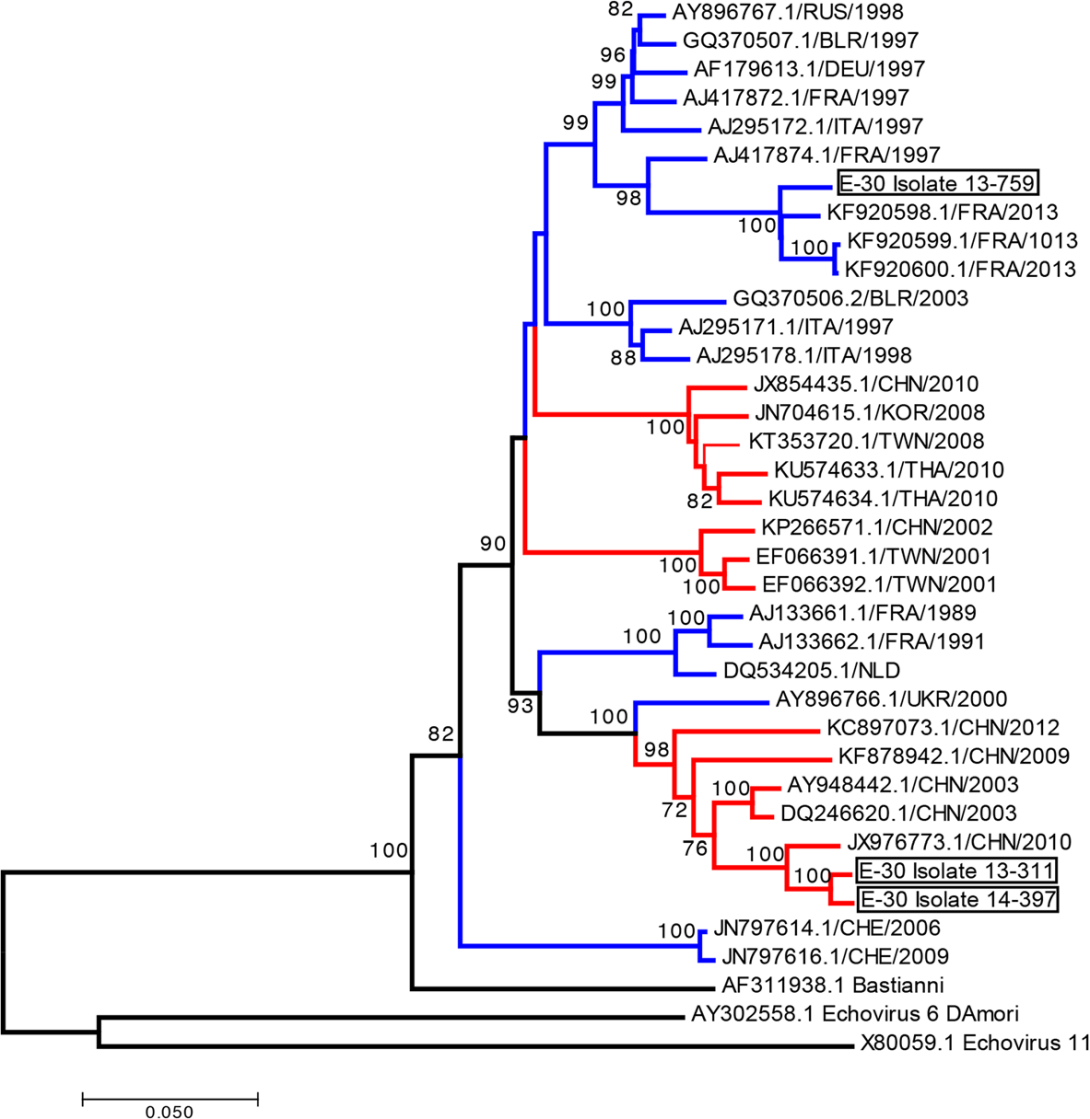
Genetic sequence alignment of E-30 structural proteins VP1–4. Phylogenetic analysis of echovirus strains based on nucleotide regions encoding for full capsid proteins using representative strains from the GenBank database. Branch colors mark isolation location (Europe, blue; Asia, red). The strains identified in this work are indicated by black rectangles. Only bootstrap values > 70% are shown at relevant nodes. Sequences of prototypes strains of Echovirus11 (X80059.1) and Echovirus 6 (AY3025588.1) were used as an outgroup. Branch length is drawn to the indicated scale (number of nucleotide substitutions per site)

The genetic relationships between the isolated strains and other E-30 strains sequences retrieved from GenBank were investigated by constructing neighbor-joining nt phylogenies for the complete P1 nucleotide regions. (Fig. 10). Both, the 13-311 and 14-397 strains belonged to a cluster, containing viruses isolated mostly in China (2003–2012), but also older European strains (1989–2000). Closest phylogenetic relationships were observed with the JX976773/CHN/2010 strain (96.6–96.7% nt identity). On the other hand, the 13-759 strain was genetically closely related to the strains that were circulating in France (96.9–97.2% nt, KF920598- KF920600/FRA/2013). With the use of RDP4 Software and several algorithms no recombination events for 13-759 could be detected (data not shown).

## Discussion

The pathogenesis of Echovirus 30 CNS infections is not fully understood. In particular, a thorough insight into the emergence, transmission dynamics and virulence of different NPEV sero- and genotypes is currently lacking [6]. Especially, shifts in genotypes seem to impact the prevalence and occurrence in meningitis outbreaks with E-30 [51].

Additionally, the BCSFB has been identified as a possible entry site for viral pathogens and leukocytes into the CNS [13, 52]. At the level of the BCSFB, barrier integrity is regulated via a complex network of TJ and AJ proteins that may limit and regulate pathogen entry and leukocyte transmigration [22, 42, 53]. During viral infection, the involvement of the proteins ZO1, ZO2, and ZO3 at the level of the TJs, and cadherins and nectins at the level of the AJs has been confirmed [53, 54].

Application of the HIBCPP cells in an in vitro cell culture model has proven to enable the investigation of E-30 infection of human choroid plexus epithelial cells mimicking the BCSFB in vivo and to analyze barrier alterations and specific immune responses after infection. In our previous work, we have shown that E-30 infects HIBCPP cells from both the basolateral and apical cell sides and infection elicits a specific chemokine expression and leukocyte migration pattern [19, 23]. In this in vitro model consisting of HIBCPP cells, we now investigated specific effects of three E-30 outbreak strains, isolated in Germany in 2013, in comparison to E-30 Bastianni to evaluate the impact of different genotypes within our model. Moreover, even though E-30 Bastianni is considered the prototype E-30 strain, we were interested in data with currently in Germany circulating genotypes.

Interestingly, infection with the three different outbreak strains led to diverse effects on the intact barrier of HIBCPP cells in vitro. Changes in barrier integrity were explicitly observed after the infection with 13-311 or E-30 Bastianni. Furthermore, a correlation of the decrease in TEER, as a parameter for barrier function, and changes in the TJ and AJ morphology, i.e., the presentation of ZO1, occludin and to a lesser extent E-cadherin, in immunofluorescence was possible. A change in the expression of TJ proteins may be of major importance for an intact barrier and TJ morphology. Western blot analyses did not show any virus-induced changes to ZO1, occludin or E-cadherin protein expression after 28 h of infection. This might be due to the overall low percentage of infected cells that allows observation of TJ alterations (e.g., degradation of associated proteins at affected cells) in the IF analysis, but not in the western blots, were all cells of the layer, including those not affected, are taken into account.

It has previously been shown that different NPEV serotypes affect the barrier of endothelial cells divergently [17]. For the BBB, investigation in brain endothelial cells (hCMEC/D3) revealed that different enterovirus serotypes caused varying effects on the barrier integrity. Cytolytic E-6, E-11, E-12, and E-30 majorly impaired the endothelial barrier, resulting in decreased TEER, increased paracellular permeability, and reduction in actin staining. In contrast, EV-A71 and coxsackievirus group B (CV-B) types had no impact on barrier integrity [17]. At the level of the BBB, in vitro studies involving other viruses could identify strain-specific effects on BBB permeability for three mouse hepatitis virus (MHV) variants during infection of immortalized mouse endothelial cells (bEnd.3). These effects were attributed to alterations in the barrier proteins ZO1, occludin, and E-cadherin on both RNA and protein levels. Despite all three serotypes that affect the barrier integrity, only the MHV3 strain was able to cross the BBB [55].

At epithelial barriers, it has been shown that CV-B3 infection of polarized epithelial cells (Caco-2 cells) led to a reduction in TEER and an increase in paracellular permeability of mannitol and dextran [56]. A direct link between viral impact on TJ morphology and changes in the TEER has also been shown in human retinal pigment epithelial (HRPE) cells after infection with HIV-1. Infection led to a decrease in TEER, which was in accordance with increased paracellular movement of sodium fluorescein. The reduced barrier integrity was linked to the downregulation of various junctional proteins, such as ZO1 and occludin [57]. In line are observations made at the airway epithelial after adenovirus infection. The decrease in TEER and rise in permeability of inulin, mannitol, and dextran was dependent on the functionality of the AJ protein E-cadherin [58]. Of note, enteroviruses have also been shown to take advantage of intercellular junctions to infect the CNS sometimes leading to alteration in protein expression [53, 59].

In response to viral entry into the CNS leukocytes migrate to the site of infection. To cross the protective barriers of the brain, leukocytes have the possibility to migrate either via the para- or transcellular route. Across endothelium, there are opposing views concerning the decisive factors for the choice of the mode of diapedesis, namely, trans- versus paracellular. On the one hand, it has been proposed in in vitro studies applying primary rat endothelial cells from the heart and brain and human lung and human microvascular endothelial cell lines that the path of least resistance is chosen by migrating leukocytes [60]. Conversely, in more recent studies applying different BBB models the barrier integrity was not decisive for the preferred pathway and rapid remodeling of TJ even in very tight endothelium was observed during paracellular transmigration [37, 61, 62]. Noteworthy, the involvement of chemokine and integrin activation can influence the pathway of leukocytes migration [60, 63], and we have recently shown, that infection of HIBCPP cells with E-30 Bastianni increased the migration of naive T-cells and PMN [23]. We hypothesized that infection with E-30 Bastianni and subsequent barrier impairment, including increased paracellular permeability, leads to a predominance of paracellular leukocyte migration. To verify this hypothesis, E-30 Bastianni was used, due to its most prominent effects on the barrier function and morphology of TJ and AJ. We could show in immunofluorescence studies as well as in TEM and FIB/SEM analysis that PMN and T-cells use both modes of diapedeses across HIBCPP cells. This paralleled our previous observations that PMN use both pathways after bacterial infection of HIBCPP cells with *Neisseria meningitidis* [39].

Most evidence in the literature for PMN transmigration pointed towards a preference of the paracellular route [64, 65]. Still, after bacterial infection of PCPEC electron microscopic imaging revealed mainly transcellular migration [38], whereas in HICBPP both pathways are used [39]. Both in vivo and in vitro it was also demonstrated at the BBB that the level of endothelial ICAM seems crucial for the pathway of diapedesis and that for T-cells, the transcellular mode of diapedesis was more common [14, 37, 66]. The current study did not analyze the role of ICAM in the regulation of transmigration, since, ICAM is apically expressed on HIBCPP cells (unpublished data), so that leukocytes primarily do not interact with this integrin ligand at first contact. Thus, its role in our model should not be critical. Interestingly, primary CD3^+^ T-cells were detected to only use the paracellular transendothelial route in vitro under flow conditions [67].

Evidence exists that barrier disruption is not a direct effect of viral infection of the CNS, but rather caused by a secondary effect such as virus replication within the brain [62]. In this context, we were able to show strain-specific growth patterns in HIBCPP cells. In this model, all three outbreak strains presented comparable percentages of infected cells, in contrast to E-30 Bastianni, which infected significantly more HIBCPP cells. Therefore, the similar effect on barrier function by strains E-30 Bastianni and 13-311 (functional and morphological impairment), and strains 13-759 and 14-397 (no observed effect), respectively, is most likely not related to viral replication levels or infection efficacy. Furthermore, the experiments on the localization of the viral genome copies within different compartments of the system indicated a faster infection frequency of E-30 Bastianni. In contrast, strain 13-311 was comparable to strain 13-759 in this aspect.

To carry out cell-line comparison studies, RD cells were used because they are highly sensitive to E30 and other enterovirus strains. No significant strain-specific difference in replication in RD cells has been observed for the three outbreak strains. We therefore concluded that the replication pattern of the different outbreak strains cannot completely account for the differences on HIBCPP TJ and AJ function and morphology.

In order to investigate the possible determinants of the variant-specific differences in growth and effects on HIBCPP cell barrier function and morphology, sequence analysis of the viral strains was carried out. The capsid of infectious E-30 virions consist of four structural proteins, VP1-VP4, out of which only VP1- VP3 are in part exposed on the surface of the capsid. The VP1 protein has highly variable solvent exposed regions that define the antigenic characteristics of the serotype [11]. Important and frequent genetic recombination enhances sporadic seasonal outbreaks and extensive epidemics worldwide [68–71]. Most noteworthy are the genetic mutations in the VP1 region of E-30, which have been associated with meningitis causing outbreak serotypes [11, 72, 73]. Even the slightest changes in the amino acid sequence can influence the affinity of a virus for a particular receptor [74]. For example, a variant-specific effect on epithelial cells was proposed to be due to minor variations in the amino acid sequence in the P1 region of clinical CV-B isolates [75], and replacing a single amino acid in a CV-B variant confers the ability to infect RD cells [76]. Similar results were obtained by Smura et al. who first investigated strain-specific tropism and went on to show that a single amino acid replacement in the VP1 region of CV-B6-Schmitt strain may lead to enhanced replication and caused the non-lytic to lytic phenotypic switch [77]. Several clusters of increased frequency of amino acid variations have been identified, but so far not directly linked to the observed differences in the strains. Instead, we report a panel of amino acid substitutions that occurred in regions encoding both structural and non-structural proteins. Although so far, none of the mutations has been directly correlated with the observed effects, it would be possible that multiple genetic factors were involved in the phenotypic consequences. Intriguingly, despite the different cellular effects in vitro, polyproteins of the more virulent 13-311 and the less virulent 14-397 strain differed in only 10 amino acids. Moreover, phylogeny revealed global circulation of these E-30 variants. The strains 13-311 and 14-397 seem closely related viruses. Software analysis of recombination did not suggest that 13-759 could be a recombinant form. Further experimental studies with site-directed mutagenesis of infectious clones are needed to determine how these specific amino acid changes contribute to the pathogenic features and widespread of the virus.

## Conclusion

We were able to show E-30 strain-specific effects on the integrity of the BCSFB in vitro. Particular 13-311 stood out, which, despite its many similarities to the other outbreak strains in relation to replication, viral dissemination or paracellular permeability, was the only one, which had similar effects on the TJ proteins of the BCSFB model as the prototype strain E-30 Bastianni. Despite the partial loss of junctional integrity induced by E-30 Bastianni, the migration of PMN and T-cells occurred both para- and transcellularly across the HIBCPP cells. The full genome sequencing highlighted genetic differences, which could, however, not be attributed to the experimentally observed differences. The study contributes to the understanding of the impact of different circulating E-30 genotypes on the level of the BCSFB. Still, for translation into the clinics, a correlation with prospectively assessed patient data is necessary. Consequently, a combination of functional in vitro and in vivo data (virulence, host cell response, and strain-specificity) and clinical data from patients, gathered during enteroviral outbreaks, could lead to a deeper understanding of enteroviral pathogenesis.

## Additional files

Additional file 1: Measurement of HIBCPP cell viability after infection with E-30 with the Live/Dead and lactate dehydrogenase (LDH) assay. (A) Live/dead assay on HIBCPP cells after 28 h of infection with E-30 Bastianni, 13-311, 13-759, and 14-397. Representative images of four independent experiments each performed in triplicates are shown. (B) LDH activity assay on HIBCPP cells after 28 h of infection. LDH-release into the supernatant by HIBCPP cells was measured after 28 h of infection with different echovirus strains. Data are shown as mean + SD of four independent experiments each performed in triplicates. Technical triplicates were used during the analytical evaluation. (TIFF 1907 kb)

Additional file 3: Paracellular PMN migration across HIBCPP in FIB/SEM-inverted images. FIB/SEM serial sections of paracellular polymorphonuclear neutrophil (PMN) migration through HIBCPP cells. Shows inverted SEM images from the condition HIBCPP+PMN + T-cells+E-30 + IL8. The video shows a paracellular migrating PMN presented in Fig. 8a, b in orthoslices. (AVI 12638 kb)

Additional file 9: Despite longer incubation periods, 13-759 and 14-397 do not show an impact on barrier integrity. Barrier integrity of HIBCPP cells was evaluated via measurement of the transepithelial electrical resistance (TEER) (A) at indicated time points after infection with E-30 Bastianni, 13-311, 13-759, or 14-397. TEER values at the start of the experiment (white bars), after 24 h (light gray) and after 48 h (dark gray) are shown.Data are shown as mean + SD of 2 independent experiments carried out in quadruples (B) Live/dead assay on HIBCPP cells after 48 h of infection with E-30 Bastianni, 13-311, 13-759, and 14-397. Representative images of two independent experiments each performed in triplicates are shown (C) HIBCPP cells were infected with E-30 Bastianni, 13-311, 13-759, and 14-397 for 48 h and ZO1 staining was compared; cell layers were stained for nuclei with DAPI (shown here in blue), VP1 (shown here in green), and ZO-1 (red). For detailed description of image acquisition and preparation, please refer to Fig. 2. Two images per strain showing different grouping of parallel staining are displayed horizontally (column one: only ZO-1; column two: DAPI, VP-1, and ZO-1; E-30 strains are listed vertically. The images shown are representative examples of multiple stainings taken from two independent experiments each performed in duplicates. (TIFF 13334 kb)

Additional file 10: Verification of virulence after E-30 passage across the HIBCPP cells. HIBCPP cells were infected with E-30 Bastianni*,* 13-311, 13-759, and 14-397 for 24 and 48 h. (A) Shows the viral genome copies (shown in copies/ml) harvested after 24 or 48 h from the lower compartment (apical cell side). A schematic representation of the experimental setup indicates the experimental procedure. The undiluted supernatant was added to confluent RD monolayers, and the cytopathic effect was observed over 24 (B) and 48 h (C). Virulence was confirmed through the RD cells detaching from the well, rounding off and finally lysing. All viral strains show to have a cytopathic effect on RD cells. The images are representative frames from 2 experiments. (TIFF 9646 kb)

Additional file 11: E-30 sequence alignments. Positions identical to those of Bastianni are indicated as dots. (A) Amino acid alignment of the P1 region. The VP4, VP3, VP2, and VP1 protein sequences are shown in red, green, blue, and purple, respectively. (B) Amino acid alignment of the P2 region. The protein 2C, 2B, and 2A sequences are shown in raspberry, orange, and light blue, respectively. (C) Amino acid alignment of the P3 region. The 3C protease, VPg, and RNA-dependent RNA polymerase sequences are shown in green, purple, and red, respectively. (D) Nucleotide alignment of 5′UTR regions. (E) Nucleotide alignment of 3′UTR regions. (PDF 3120 kb)

Additional file 12: Amino acid substitutions observed between E-30 Bast. and the outbreak strains. To illustrate differences in between the E-30 strains used, a table was designed with the data that has already been displayed in Additional file 11. The positions that matched between 13-311 and the other three E-30 strains are highlighted in green; those that were different are left blank (white). 13-311 and 14-397 vary in 10 amino acids, whereas 13-311 and 13-759 vary in 70 amino acids. (PDF 139 kb)

## Abbreviations

AJ: Adherens junction
BBB: Blood–brain barrier
BCSFB: Blood–cerebrospinal fluid barrier
BSA: Bovin serum albumin
CNS: Central nervous system
CSF: Cerebrospinal fluid
CV-B: Coxsackievirus B, family *Picornaviridae*, genus Enterovirus, species Human enterovirus B
E: Echovirus, family *Picornaviridae*, DAPI, 4′-6-diamidino-2-phenylindole dihydrochloride
FCS: Fetal calf serum, genus Enterovirus, species Human enterovirus B
FIB: Focused ion beam
HBMEC: Human brain microvascular endothelial cells
HIBCPP: Human choroid plexus papilloma cells
Hr: Hour
HRP: Horseradish peroxide
LDH: Lactate dehydrogenase
MAM: Migration assay medium
MOI: Multiplicity of infection
NPEV: Non-polio enterovirus
NRC PE: National Reference Centre for Poliomyelitis and Enteroviruses
Nt: Nucleotide
PCPEC: Primary porcine choroid plexus epithelial
PCR: Polymerase chain reaction
PMN: Polymorphonuclear neutrophil
qPCR: Quantitative real-time polymerase chain reaction
RD: Human rhabdomyosarcoma cells
RIPA: Radioimmunoprecipitation assay
RT: Room temperature
SD: Standard deviation
SEM: Scanning electron microscopy
TEER: Transepithelial electrical resistance
TEM: Transmission electron microscopy
TJ: Tight junction
VP1: Viral protein 1
ZO: Zonula occludens
