# Supplementary figures and images for: Strain-dependent effects of clinical echovirus 30 outbreak isolates at the blood-CSF barrier
Source: J Neuroinflammation. 2018 Feb 20;15:50. doi: 10.1186/s12974-018-1061-4 (PMC5819246; doi:10.1186/s12974-018-1061-4)

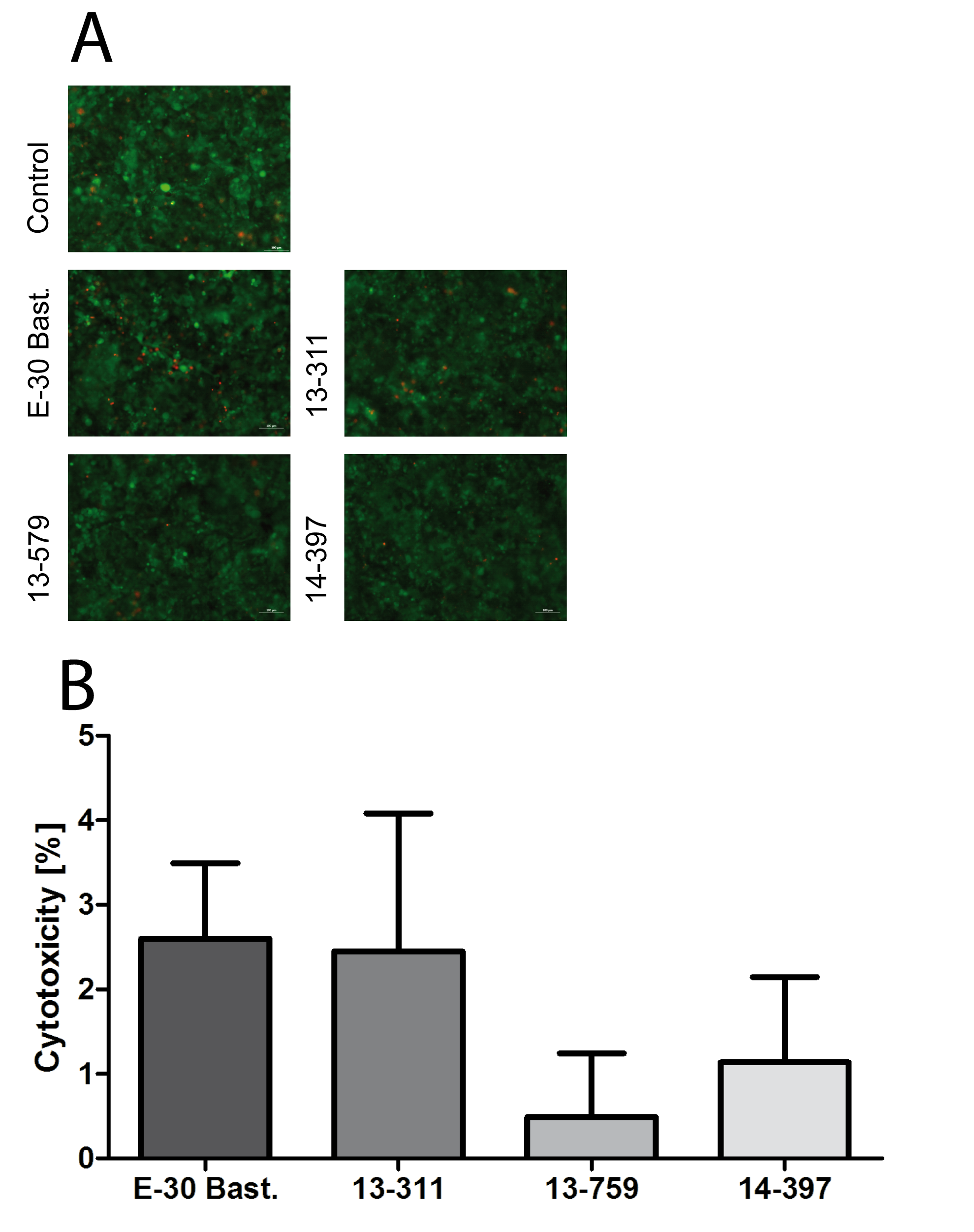

Supplement: Supplementary file 7 — Measurement of HIBCPP cell viability after infection with E-30 with the Live/Dead and lactate dehydrogenase (LDH) assay. (A) Live/dead assay on HIBCPP cells after 28 h of infection with E-30 Bastianni, 13-311, 13-759, and 14-397. Representative images of four independent experiments each performed in triplicates are shown. (B) LDH activity assay on HIBCPP cells after 28 h of infection. LDH-release into the supernatant by HIBCPP cells was measured after 28 h of infection with different echovirus strains. Data are shown as mean + SD of four independent experiments each performed in triplicates. Technical triplicates were used during the analytical evaluation. (TIFF 1907 kb) [file 12974_2018_1061_MOESM1_ESM.tif]

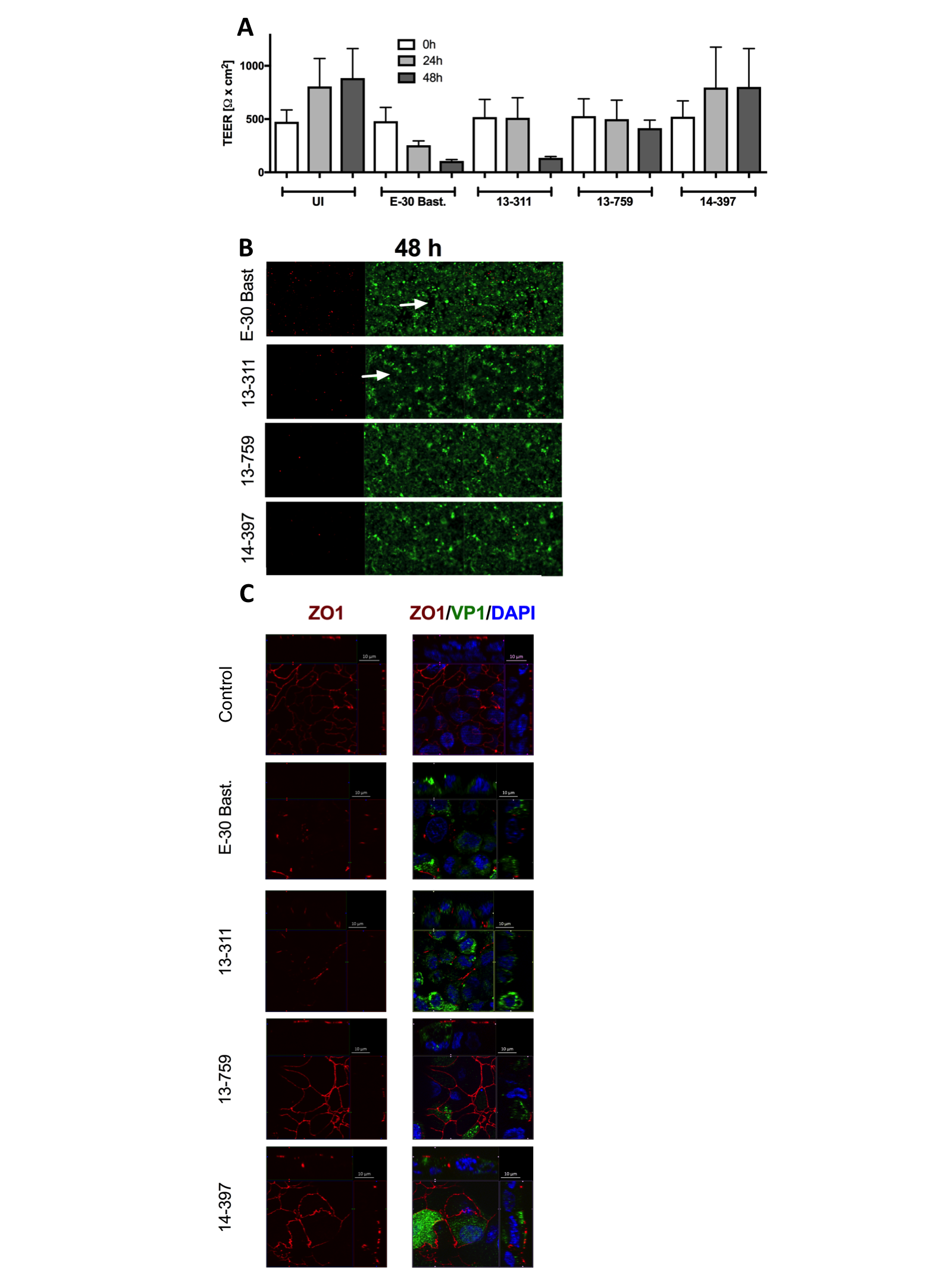

Supplement: Supplementary file 9 — Despite longer incubation periods, 13-759 and 14-397 do not show an impact on barrier integrity. Barrier integrity of HIBCPP cells was evaluated via measurement of the transepithelial electrical resistance (TEER) (A) at indicated time points after infection with E-30 Bastianni, 13-311, 13-759, or 14-397. TEER values at the start of the experiment (white bars), after 24 h (light gray) and after 48 h (dark gray) are shown.Data are shown as mean + SD of 2 independent experiments carried out in quadruples (B) Live/dead assay on HIBCPP cells after 48 h of infection with E-30 Bastianni, 13-311, 13-759, and 14-397. Representative images of two independent experiments each performed in triplicates are shown (C) HIBCPP cells were infected with E-30 Bastianni, 13-311, 13-759, and 14-397 for 48 h and ZO1 staining was compared; cell layers were stained for nuclei with DAPI (shown here in blue), VP1 (shown here in green), and ZO-1 (red). For detailed description of image acquisition and preparation, please refer to Fig. 2. Two images per strain showing different grouping of parallel staining are displayed horizontally (column one: only ZO-1; column two: DAPI, VP-1, and ZO-1; E-30 strains are listed vertically. The images shown are representative examples of multiple stainings taken from two independent experiments each performed in duplicates. (TIFF 13334 kb) [file 12974_2018_1061_MOESM9_ESM.tif]

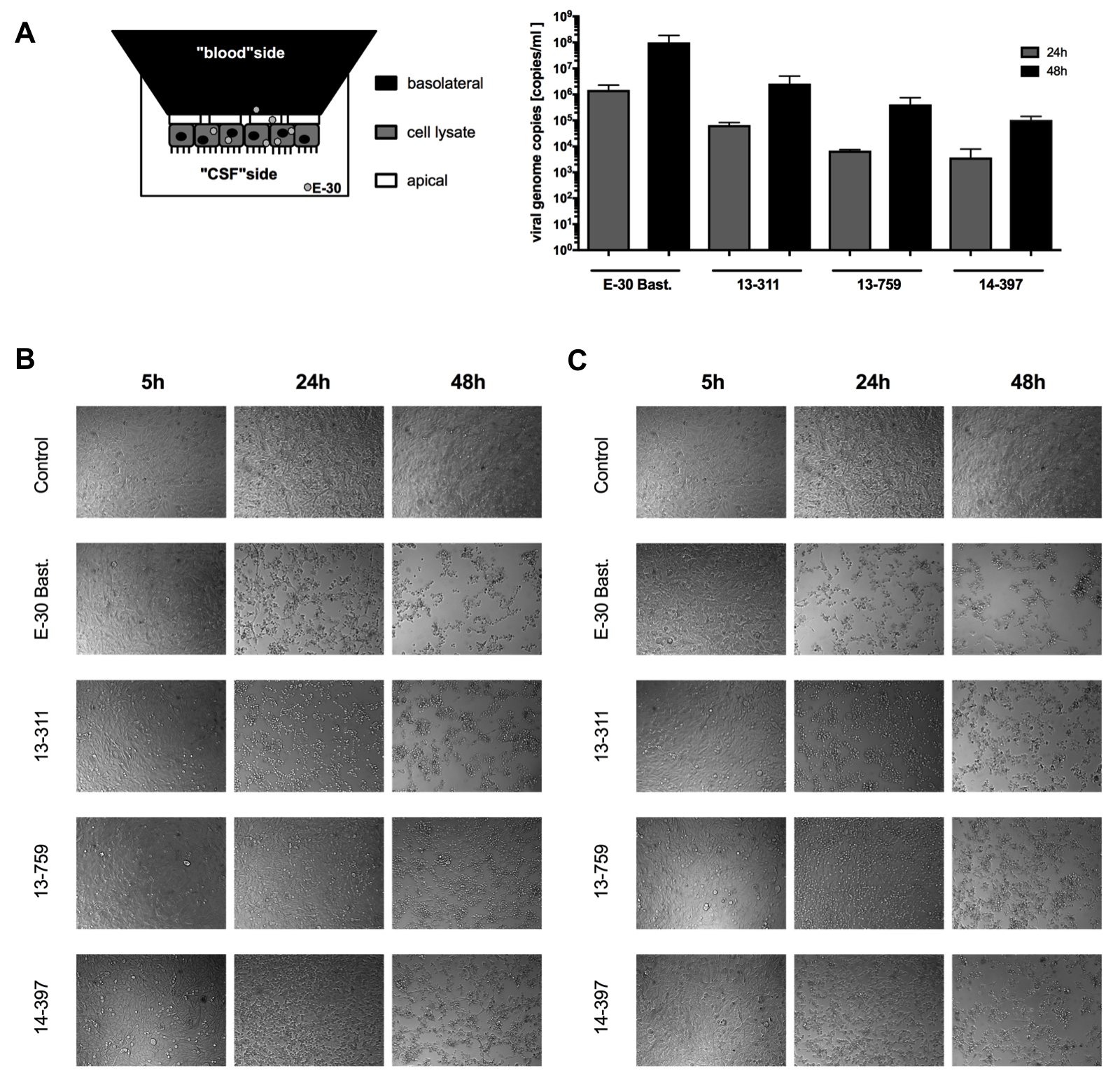

Supplement: Supplementary file 10 — Verification of virulence after E-30 passage across the HIBCPP cells. HIBCPP cells were infected with E-30 Bastianni, 13-311, 13-759, and 14-397 for 24 and 48 h. (A) Shows the viral genome copies (shown in copies/ml) harvested after 24 or 48 h from the lower compartment (apical cell side). A schematic representation of the experimental setup indicates the experimental procedure. The undiluted supernatant was added to confluent RD monolayers, and the cytopathic effect was observed over 24 (B) and 48 h (C). Virulence was confirmed through the RD cells detaching from the well, rounding off and finally lysing. All viral strains show to have a cytopathic effect on RD cells. The images are representative frames from 2 experiments. (TIFF 9646 kb) [file 12974_2018_1061_MOESM10_ESM.tiff]
