# Supplementary material for: Strain-dependent effects of clinical echovirus 30 outbreak isolates at the blood-CSF barrier
Source: J Neuroinflammation. 2018 Feb 20;15:50. doi: 10.1186/s12974-018-1061-4 (PMC5819246; doi:10.1186/s12974-018-1061-4)
Supplement: Supplementary file 11 — E-30 sequence alignments. Positions identical to those of Bastianni are indicated as dots. (A) Amino acid alignment of the P1 region. The VP4, VP3, VP2, and VP1 protein sequences are shown in red, green, blue, and purple, respectively. (B) Amino acid alignment of the P2 region. The protein 2C, 2B, and 2A sequences are shown in raspberry, orange, and light blue, respectively. (C) Amino acid alignment of the P3 region. The 3C protease, VPg, and RNA-dependent RNA polymerase sequences are shown in green, purple, and red, respectively. (D) Nucleotide alignment of 5′UTR regions. (E) Nucleotide alignment of 3′UTR regions. (PDF 3120 kb) [file 12974_2018_1061_MOESM11_ESM.pdf]

A

```
                   10        20        30        40        50        60        70        80        90        100
          ---------+---------+---------+---------+---------+---------+------VP4+---------+---------+---------+
Bastianni MGAQVSTQKTGAHETGLSASGNSVIHYTNINYYKDSASNSLNRQDFTQDPSRFTEPVKDVMIKTLPALNSPTVEECGYSDRVRSITLGNSTITTQECANV
13311     ...............S.N...S.I......................S....K................................................
13759     ...................................................K.........................F......................
14397     ..............SS.N...S.I...........................K................................................

                   110       120       130       140       150       160       170       180       190       200
          ---------+---------+---------+---------+---------+---------+---------+---------+---------+---------+
Bastianni VVGYGVWPTYLSDHEATAVDQPTQPDVATCRFYTLESVKWESSSAGWWWKFPEALSDMGLFGQNMQYHYLGRAGYTIHVQCNASKFHQGCLLVVCVPEAE
13311     .............N..........................................................T...........................
13759     .....E.......R............................A.............................S...........................
14397     .............N..........................................................T...........................

                   210       220       230       240       250       260       270       280       290       300
          ---------+---------+---------+---------+---------+---------+---------+---------+---------+---------+
Bastianni MGAATTDHAMNHTKLSNIGQAMEFSAGKSTDQTGPQTAVHNAGMGVAVGNLTIYPHQWINLRTNNSATIVMPYINSVPMDNMYRHYNFTLMVIPFAKLEH
13311     ..........................E..........................F..............................................
13759     ..........................E..........................F..............................................
14397     .....................................................F..............................................

                   310       320       330       340       350       360       370       380       390       400
          ---------+---------+---------VP2-------+---------+---------+---------+---------+---------+---------+
Bastianni SPQASTYVPITVTVAPMCAEYNGLRLAGHQGLPTMNTPGSTQFLTSDDFQSPSAMPQFDVTPEIQIPGQVRNLMEIAEVDSVVPVNNTEEHVNSIEAYRI
13311     ...........................................................................V.............GN...M.....
13759     ....................................................................................................
14397     ...........................................................................V.............GN...M.....

                   410       420       430       440       450       460       470       480       490       500
          ---------+---------+---------+---------+---------+---------+---------+---------+---------+---------+
Bastianni PVRPQTNSGEQVFGFQLRPGYDSVLKHTLLGEILNYYANWSGSMKLTFMYCGAAMATGKFLIAYSPPGAGVPGSRKDAMLGTHVIWDVGLQSSCVLCVPW
13311     ......S..........Q..H.........................................T.....................................
13759     ......S..........Q........R..................................M......................................
14397     ......S..........Q..H...............................................................................

                   510       520       530       540       550       560       570       580       590       600
          ---------+---------+---------+---------+---------+---------+----VP3--+---------+---------+---------+
Bastianni ISQTNYRYVTSDAYTDAGYITCWYQTSIVTPPDIPTTSTILCFVSACNDFSVRLLRDTPFITQQALYQNDPEGALNKAVGRVADTIASGPVNTEQIPALT
13311     ........................................................................S.I.R........V..............
13759     ........................................................................S.I.R........V..............
14397     ............V...........................................................S.I.RT.......V..............

                   610       620       630       640       650       660       670       680       690       700
          ---------+---------+---------+---------+---------+---------+---------+---------+---------+---------+
Bastianni AVETGHTSQVVPSDTMQTRHVVNFHTRSESSLENFMGRAACVYIAHYATEKANDDLDRYTNWEITTRQVAQLRRKLEMFTYMRFDLEITFVITSSQRTSN
13311     .....................I.Y.......I.............Q.....V..E..................................L.........T
13759     .......................Y.......I.............Q.....V..E............................................T
14397     .....................I.Y.......I.............Q.....V..E..................................L.........T

                   710       720       730       740       750       760       770       780       790       800
          ---------+---------+---------+---------+---------+---------+---------+---------+---------+---------+
Bastianni RYASDSPPLTHQIMYVPPGGPIPTGYEDFAWQTSTNPSVFWTEGNAPPRMSIPFMSVGNAYCNFYDGWSHFSQSGVYGYTTLNNMGHLYFRHVNKSTGYP
13311     T...........V..........KS........................................................................A..
13759     T...........V..........RS........................................................................A..
14397     T...........V..........KS........................................................................A..

                   810       820       830       840       850       860
          ---------+---------+---------+---------+---------+------VP1+
Bastianni VNSVARVYFKPKHVKAWVPRAPRLCPYLYAKNVNFDVQGVTESRGKITLDRSTHNPVLTT
13311     ..............................R.............D...........LTN.
13759     ........................................................L.N.
14397     .........................Q....R.............D...........LTN.
```

B

```
                   870       880       890       900
           ---------+---------+---------+---------+
Bastianni  GAFEQQSGAAYVGNYRLVNRHLATHTDWQNCVWEDYNRDL
13311      .V.G............V........N..............
13759      .V.G.....V......V.......................
14397      .V.G............V........N..............

                   910       920       930       940       950       960       970       980       990       1000
           ---------+---------+---------+---------+---------+---------+---------+---------+---------+---------+
Bastianni  LVSTTTAHGCDTIARCQCTTGVYFCASRNKHYPVTFEGPGLVEVQESEYYPKRYQSHVLLAAGFSEPGDCGGILRCEHGVIGLVTMGGEGVVGFADVRDL
13311      ...........V......S...............S.................................................................
13759      ..................................N............................................A....................
14397      ...........V......S...............S.................................................................

                   1010      1020      1030      1040      1050      1060      1070      1080      1090      1100
           ---2C----+---------+---------+---------+---------+---------+---------+---------+---------+---------+
Bastianni  LWLEDDAMEQGVKDYVEQLGNAFGSGFTNQICEQVNLLKESLIGQDSILEKSLKALVKIISALVIVVRNHDDLITVTATLALIGCTTSPWRWLKHKVSQY
13311      ..........................................V.............I................V........V...S.......Q.....
13759      ............R.............................V.....................V.....................A.......Q.T...
14397      ..........................................V.............I................V........V...S.......Q.....

                   1110      1120      1130      1140      1150      1160      1170      1180      1190      1200
           -------2B-+---------+---------+---------+---------+---------+---------+---------+---------+---------+
Bastianni  YGIPMAERQNNNWLKKFTEMTNACKGMEWIAIKIQKFIEWLKVKILPEVKEKHEFLNRLKQLPLLESQIATIEQSAPSQSDQEQLFSNVQYFAHYCRKYA
13311      ...L.......G...................N.................R......T...........................................
13759      ...........G.....................................R..................................................
14397      ...........G...................N.................R......T...........................................

                   1210      1220      1230      1240      1250      1260      1270      1280      1290      1300
           ---------+---------+---------+---------+---------+---------+---------+---------+---------+---------+
Bastianni  PLYAAEAKRVFSLEKKMSNYIQFKSKCRIEPVCLLLHGSPGAGKSVATNLIGRSLAEKLNSSVYSLPPDPDHFDGYKQQAVVIMDDLCQNPDGRDVSLFC
13311      ....T........................................................................................K......
13759      .................G...........................................................................K......
14397      ....T........................................................................................K......

                   1310      1320      1330      1340      1350      1360      1370      1380      1390      1400
           ---------+---------+---------+---------+---------+---------+---------+---------+---------+---------+
Bastianni  QMVSSVDFVPPMAALEEKGILFTSPFVLASTNAGSINAPTVSDSRALARRFHFDMNIEVISMYSQNGKINMPMSVKTCDEECCPVNFKRCCPLVCGKAIQ
13311      ........................................................................................K...........
13759      ....................................................................................A...K....L......
14397      ........................................................................................K...........

                   1410      1420      1430
           ---------+---------+---------+------2A
Bastianni  FIDRRTQVRYSLDMLVTEMFREYNHRHSVGATLEALF
13311      ....K................................
13759      .......................T.............
14397      ....K................................
```

C

```
            1440      1450      1460      1470      1480      1490      1500
          --+---------+---------+---------+---------+---------+---------+
Bastianni QGPPVYREIKISVAPETPPPPAIADLLKSVDSEAVREYCKEKGWLVPEINSTLQIEKHVSRAF
13311     .........................................R.....................
13759     .............T..................................V..............
14397     .........................................R.....................

                   1510      1520      1530      1540      1550      1560      1570      1580      1590      1600
          ---------+---------+--Pro3C--+---------+---VPg---+---------+---------+----RdRp--+---------+---------+
Bastianni ICLQALTTFVSVAGIIYIIYKLFAGFQGAYSGMPNQKSKVPTLRQAKVQGPAFEFAVAMMKRNASTVKTEYGEFTMLGIYDRWAVLPRHAKPGPTIIMND
13311     ..............................T......P.........................S..........................R.....L...
13759     ..............................T......P............................AR............................L...
14397     ..............................T......P.........................S..........................R.....L...

                   1610      1620      1630      1640      1650      1660      1670      1680      1690      1700
          ---------+---------+---------+---------+---------+---------+---------+---------+---------+---------+
Bastianni QEVGVVDAKELVDKDGTNLELTLLKLNRNEKFRDIRGFLAREEAEVNEAVLAINTSKFPNMYIPVGQVTDYGFLNLGGTPTKRMLMYNFPTRAGQCGGVL
13311     .....L..................................K..V........................................................
13759     .....L.....................................V..................................A.....................
14397     .....L..................................K..V........................................................

                   1710      1720      1730      1740      1750      1760      1770      1780      1790      1800
          ---------+---------+---------+---------+---------+---------+---------+---------+---------+---------+
Bastianni MSTGKVLGVHVGGNGHQGFSAALLRHYFNDEQGEIEFIESSKEAGFPVINTPSKTKLEPSVFHHVFEGNKEPAVLRNGDPRLKANFEEAIFSKYIGNVNT
13311     ........I...............K.................D....................Q...................V................
13759     ........I...............K....E...........RD....................Q....................................
14397     ........I...............K.................D....................Q...................V................

                   1810      1820      1830      1840      1850      1860      1870      1880      1890      1900
          ---------+---------+---------+---------+---------+---------+---------+---------+---------+---------+
Bastianni HVDEYMMEAVDHYAGQLATLDISTEPMKLEDAVYGTEGLEALDLTTSAGYPYVALGIKKRDILSKKTKDLAKLKECMDKYGLNLPMVTYVKDELRSAEKV
13311     ......................................................................T.........................VD..
13759     ......L............................................................R..T.........................T...
14397     .........I............................................................T.........................VD..

                   1910      1920      1930      1940      1950      1960      1970      1980      1990      2000
          ---------+---------+---------+---------+---------+---------+---------+---------+---------+---------+
Bastianni AKGKSRLIEASSLNDSVAMRQTFGNLYKTFHMNPGIVTGSAVGCDPDLFWSKIPVMLDGHLIAFDYSGYDASLSPVWFACLKLLLEKLGYSHKETNYIDY
13311     ...............................L....................................................................
13759     ...............................L...............V.....................................G...TN........
14397     ...............................L....................................................................

                   2010      2020      2030      2040      2050      2060      2070      2080      2090      2100
          ---------+---------+---------+---------+---------+---------+---------+---------+---------+---------+
Bastianni LCNSHHLYRDKHYFVRGGMPSGCSGTSIFNSMINNIIIRTLMLKVYKGIDLDQFRMIAYGDDVIASYPHPIDASLLAEAGKGYGLIMTPADKGECFNEVT
13311     ....................................................................W...............................
13759     ....................................................................W............D..................
14397     ....................................................................W...............................

                   2110      2120      2130      2140      2150      2160      2170      2180      2190
          ---------+---------+---------+---------+---------+---------+---------+---------+------+RdRp---
Bastianni WTNVTFLKRYFRADEQYPFLVHPVMPMKDIHESIRWTKDPKNTQDHVRSLCLLAWHNGEQEYEEFVSKIRSVPVGRCLTLPAFSTLRRKWLDSF
13311     .A.........................................................H.....IQ...........................
13759     ...........................................................H.....IR............I..............
14397     .A..................I......................................H.....IQ...........................
```

D

```
                   10        20        30        40        50        60        70        80        90       100
          ---------+---------+---------+---------+---------+---------+---------+---------+---------+---------+
Bastianni ------------------------ACCCACCCACAGGGCCCACTGGGCGCCAGCACACTGGTATCGTGGTACCTTTGTGCGCCTGTTTTATACCCCCTAC
13311     --TTAAAACAAGCCTGTGGGTTGA......T...........A.......T..............AC........................C.A....TT
13759     TTTTAAAACAAGC-TGTGGGTTGAT.........................T..T...........AC.......................CCTA...CT-
14397     -TTTAAAACAAGCCTGTGGGTTGA..................A.......T..............AC........................C.A....T-

                  110       120       130       140       150       160       170       180       190       200
          ---------+---------+---------+---------+---------+---------+---------+---------+---------+---------+
Bastianni CCCCACACGTACTCTAGAAGCAAGGCAAACCTGACCAATAGTAGGTGTGGCACACCAGCCGCATTTTGGTCAAGCACTTCTGTTTCCCCGGACCGAGTAT
13311     ....GA...A.ACT......TTT.AATT.AAC.GT......G.AACCCA.T......A.T.GGC.G..AC.............GA...............
13759     ....GA.A.A.ACT.......TCAA.TT.TG..GT........A.CTCA.T..G...A.T.AGCCAC.AC.............CG........T......
14397     ....GA...A.ACT......TTT.AATT.AAC.GT......G.AACCCA.T......A.T.GGC.G..AC.............GA...............

                  210       220       230       240       250       260       270       280       290       300
          ---------+---------+---------+---------+---------+---------+---------+---------+---------+---------+
Bastianni CAATAAGCTGCTCACGCGGCTGAAGGAGAAACCGTTCGTTACCCGACCAGCTACTTCGAGAAACCTAGTAACACTATGAACGTTGCGGAGTGTTTCGTTC
13311     .........A......T..............A.............G...AT.............T.....C...C.....A......C..C......C..
13759     ................T..............A.........T...G...AT..............C....C...C.....A......CG.C......C..
14397     .........A......T..............A.............G...AT.............T.....C...C.....A......C..C......C..

                  310       320       330       340       350       360       370       380       390       400
          ---------+---------+---------+---------+---------+---------+---------+---------+---------+---------+
Bastianni AGCACTTCCCCGTGTAGATCAGGTCGATGAGTCACCGCATTCCTCACGGGTGACCGTGGCGGTGGCTGCGTTGGCGGCCTGCCTACGGGTTCGCCCGTA
13311     C....GA....A............CT.............G.A..C.......................................CTT...GCAA...AAG
13759     C....AA....A.........G..C..............G....C......C.............A..................C.T..AGCAAT..A.G
14397     C....GA....A............C..............G.A..C.......................................CTT...GCAA...AAG

                  410       420       430       440       450       460       470       480       490       500
          ---------+---------+---------+---------+---------+---------+---------+---------+---------+---------+
Bastianni GGACGCTCTAATACCGACATGGTGTGAAGAGTCCATTGAGCTAGCTGGTAGTCCTCCGGCCCCTGAATGCGGCTAATCCTAACTGCGGAGCAGGTGCTCA
13311     .......TC.....T.........C........T.........AT..................................C.............A.A.C..
13759     .......TC.....T.........C........T.........AT................................................A.A.C..
14397     .......TC.....T.........C........T.........AT..................................C.............A.A.C..

                  510       520       530       540       550       560       570       580       590       600
          ---------+---------+---------+---------+---------+---------+---------+---------+---------+---------+
Bastianni CAGACCAGTGAGTAGCCTGTCGTAACGGGCAACTCTGCAGCGGAACCGACTACTTTGGGTGTCCGTGTTTCCTTTTTTTTTTCTCTTATATTGGCTGCTT
13311     ..T.......G.C..T.........T.................................................A-----.T.....C.C.........
13759     ..C.......G.C..T..................TC.......................................------.A.T.....C.........
14397     ..T.......G.C..T.........T.................................................A-----.T.....C.C.........

                  610       620       630       640       650       660       670       680       690       700
          ---------+---------+---------+---------+---------+---------+---------+---------+---------+---------+
Bastianni ATGGTGACAATTAAAGAATTGTTACCATATAGCTATTGGATTGGCCATCCGGTGACGAGCAGAGCCATTGTTTACCTCTTTGTTGGATTTGTACCTTTGA
13311     ............G.GAG......G..............................T.T.AT.....A...A.C.....T............A.T..AC.T.
13759     ............G.GAG.....................................T.C.A......G..CA.....T.G...........C..G..C....
14397     ............G.GAG......G..............................T.T.AT.....A...A.C.....T............A.T..AC.T.

                  710       720       730       740       750
          ---------+---------+---------+---------+---------+--------
Bastianni ACCACAAAGTCTTGAATACCATTCATCTCATTTTAAAGTTCAACTCAGCTAAAAGAAA
13311     ..T.T....CAC.C..A..AC..ATATA....G..G.TC.....A....A..
13759     ..T......CAC.CCTA..AC..A.ATA....G..G.CC.....A....A..
14397     ..T.T....CAC.C..A..AC..ATATA....G..G.TC.....A....A..
```

E

```
                   10        20        30        40        50        60        70        80        90       100
          ---------+---------+---------+---------+---------+---------+---------+---------+---------+---------+----
Bastianni -ATTAGAGTACAATTATATAATTTTAATTTTAATTGGCTTAACCCTACCACACTTACCGAACTAGACAACGGTGTGGTAGGGGTAAATTCTCCGCA
13311     -.......-.......A.C.----......G.....................ACC...........T.............................TTCGGTGA
13759     A.......C.....C.GTG.------...ACG.....T.C.........G..TGA........T....CAA...C.....................TTCGGTG
14397     -.......-.......A.C.----......G.....................ACC...........T.............................TTCGGTGA
```
