# Supplementary material for: Strain-dependent effects of clinical echovirus 30 outbreak isolates at the blood-CSF barrier
Source: J Neuroinflammation. 2018 Feb 20;15:50. doi: 10.1186/s12974-018-1061-4 (PMC5819246; doi:10.1186/s12974-018-1061-4)
Supplement: Supplementary file 12 — Amino acid substitutions observed between E-30 Bast. and the outbreak strains. To illustrate differences in between the E-30 strains used, a table was designed with the data that has already been displayed in Additional file 11. The positions that matched between 13-311 and the other three E-30 strains are highlighted in green; those that were different are left blank (white). 13-311 and 14-397 vary in 10 amino acids, whereas 13-311 and 13-759 vary in 70 amino acids. (PDF 139 kb) [file 12974_2018_1061_MOESM12_ESM.pdf]

#	Gene	Position	Aminoacids			
			E-30 Bast.	13311	13759	14197
1	**VP4**	15	T	T	T	S
2		16	G	S	G	S
3		18	S	N	S	N
4		22	N	S	N	S
5		24	V	I	V	I
6		47	T	S	T	T
7		52	R	K	K	K
8	**VP2**	78	Y	Y	F	Y
9		106	V	V	E	V
10		114	H	N	R	N
11		143	S	S	A	S
12		173	A	T	S	T
13		227	G	E	E	G
14		254	Y	F	F	F
15	**VP3**	376	I	V	I	V
16		390	E	G	E	G
17		391	H	N	H	N
18		395	I	M	I	M
19		407	N	S	S	S
20		418	R	Q	Q	Q
21		421	Y	H	Y	H
22		427	H	H	R	H
23		462	I	I	M	I
24		463	A	T	A	A
25		513	A	A	A	V
26	**VP1**	573	G	S	S	S
27		575	L	I	I	I
28		577	K	R	R	R
29		578	A	A	A	T
30		586	I	V	V	V
31		622	V	I	V	I
32		624	F	Y	Y	Y
33		632	L	I	I	I
34		646	H	Q	Q	Q
35		652	A	V	V	V
36		655	D	E	E	E
37		690	F	L	F	L
38		700	N	T	T	T
39		701	R	T	T	T

40		713	I	V	V	V
41		724	T	K	R	K
42		725	G	S	S	S
43		798	G	A	A	A
44		826	P	P	P	Q
45		831	K	R	K	R
46		845	G	D	G	D
47		857	V	L	L	L
48		858	V	T	L	T
49		859	T	N	N	N
50	**2C**	862	A	V	V	V
51		864	E	G	G	G
52		870	A	A	V	A
53		877	L	V	V	V
54		886	T	N	T	N
55		912	T	V	T	V
56		919	T	S	T	S
57		935	T	S	N	S
58		980	V	V	A	V
59	**2B**	1013	K	K	R	K
60		1043	I	V	V	V
61		1057	V	I	V	I
62		1065	I	I	V	I
63		1074	I	V	I	V
64		1083	I	V	I	V
65		1087	T	S	A	S
66		1095	H	Q	Q	Q
67		1097	V	V	T	V
68		1104	P	L	P	P
69	**2A**	1112	N	G	G	G
70		1132	I	N	I	N
71		1150	K	R	R	R
72		1157	N	T	N	T
73		1205	A	T	A	T
74		1218	S	S	G	S
75		1294	R	K	K	K
76		1385	V	V	A	V
77		1389	R	K	K	K
78		1394	V	V	L	V
79		1405	R	K	R	K
80		1424	N	N	T	N

81	**3A**	1451	A	A	T	A
82		1479	K	R	K	R
83		1486	I	I	V	I
84	**VPg**	1531	S	T	T	T
85		1538	S	P	P	P
86	**3C**	1564	A	S	A	S
87		1567	V	V	A	V
88		1568	K	K	R	K
89		1591	K	R	K	R
90		1597	I	L	L	L
91		1606	V	L	L	L
92		1641	R	K	R	K
93		1644	A	V	V	V
94		1679	T	T	A	T
95		1710	V	I	I	I
96		1725	R	K	K	K
97		1730	D	D	E	D
98	**RdRp**	1742	K	K	R	K
99		1743	E	D	D	D
100		1764	H	Q	Q	Q
101		1784	A	V	A	V
102		1807	M	M	L	M
103		1810	V	V	V	I
104		1868	K	K	R	K
105		1871	A	T	T	T
106		1897	A	V	T	V
107		1898	E	D	E	D
108		1932	M	L	L	L
109		1948	L	L	V	L
110		1986	E	E	G	E
111		1991	S	S	T	S
112		1992	H	H	N	H
113		2069	H	W	W	W
114		2082	G	G	D	G
115		2102	T	A	T	A
116		2121	V	V	V	I
117		2160	Q	H	H	H
118		2166	V	I	I	I
119		2167	S	Q	R	Q
120		2180	L	L	I	L
